# Neural dynamics and architecture of the heading direction circuit in zebrafish

**DOI:** 10.1038/s41593-023-01308-5

**Published:** 2023-04-24

**Authors:** Luigi Petrucco, Hagar Lavian, You Kure Wu, Fabian Svara, Vilim Štih, Ruben Portugues

**Affiliations:** 1 Institute of Neuroscience, Technical University of Munich, Munich, Germany; 2 Graduate School of Systemic Neurosciences, Ludwig-Maximilian University, Munich, Germany; 3 Department of Computational Neuroethology, Max Planck Institute for Neurobiology of Behavior – caesar, Bonn, Germany; 4 Vara, Berlin, Germany; 5 Munich Cluster of Systems Neurology (SyNergy), Munich, Germany

**Keywords:** Navigation, Neural encoding, Neural circuits

## Abstract

Animals generate neural representations of their heading direction. Notably, in insects, heading direction is topographically represented by the activity of neurons in the central complex. Although head direction cells have been found in vertebrates, the connectivity that endows them with their properties is unknown. Using volumetric lightsheet imaging, we find a topographical representation of heading direction in a neuronal network in the zebrafish anterior hindbrain, where a sinusoidal bump of activity rotates following directional swims of the fish and is otherwise stable over many seconds. Electron microscopy reconstructions show that, although the cell bodies are located in a dorsal region, these neurons arborize in the interpeduncular nucleus, where reciprocal inhibitory connectivity stabilizes the ring attractor network that encodes heading. These neurons resemble those found in the fly central complex, showing that similar circuit architecture principles may underlie the representation of heading direction across the animal kingdom and paving the way to an unprecedented mechanistic understanding of these networks in vertebrates.

## Abstract (web summary)

In this study, Petrucco, Lavian et al. identify a circuit in the hindbrain of larval zebrafish that persistently encodes heading direction. Neurons of this network, of stereotypical morphology, inhibit each other to support ring attractor dynamics.

## Main

In many animals, effective navigation in the world involves the use of cognitive maps that provide a representation of position and orientation with respect to the environment. Whereas position in space is encoded in place cells and grid cells in the mammalian hippocampal and entorhinal circuits^1^, allocentric orientation is represented by head direction cells, which are neurons that are active any time the animal faces a particular direction in space.

Head direction cells were originally described in the postsubicular cortex^2^, but have since been observed in several other cortical and subcortical areas (reviewed in Taube^3^). The activity in these head direction networks can be understood in terms of ring attractor networks, where local recurrent excitation is combined with long-range, out-of-phase inhibition to create a stable, localized bump of activity that encodes direction. This model received remarkable empirical validation with the observation of heading direction representations in the insect central complex, where key components of a ring attractor network were mapped onto its neuronal architecture^4–9^. However, such mechanistic understanding in vertebrates is still lacking.

The lowest region of the vertebrate brain in which head direction-related signals have been found is the dorsal tegmental nucleus (DTN)^10^, a paired, GABAergic nucleus located in the brainstem that originates from rhombomere 1 (ref. ^11^). In rodents, the DTN is reciprocally connected with the interpeduncular nucleus (IPN), a hindbrain structure indirectly implicated in spatial navigation^11,12^ and in the generation of heading direction representations^13^. Additionally, recent studies in larval zebrafish suggest an important role for the IPN in directional behavior^14,15^. Therefore, we leveraged the optical accessibility of the larval zebrafish as a model organism to comprehensively image the anterior hindbrain (aHB) of this vertebrate to identify any potential network activity that could be involved in the encoding of heading direction.

Using a combination of volumetric lightsheet imaging, two-photon imaging and electron microscopy (EM), we discover a circuit contained within rhombomere 1 that represents heading direction by a persistent and localized bump of activity. This activity profile smoothly translates across the neuronal population as the fish turns, mimicking the compass neurons in the central complex of insects. Furthermore, we show that this inhibitory network in the aHB forms highly organized reciprocal connections in the dorsal IPN (dIPN). This architecture is in concordance with the connectivity scheme required by ring attractor models of head direction networks^16,17^ and can provide the substrate for a cognitive map in this vertebrate brain.

## Results

### Ring attractor dynamics in the fish aHB

**Fig. 1:**
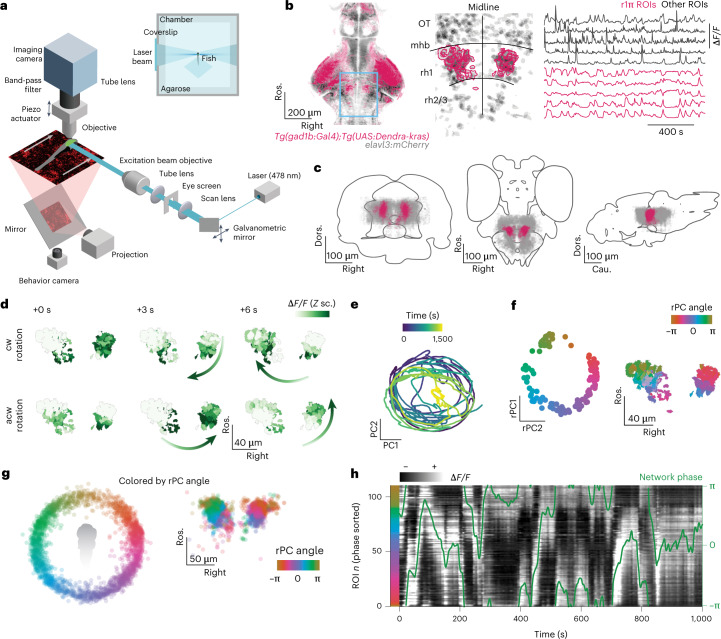
A GABAergic network in the aHB of the larval zebrafish exhibits stable circular dynamics. **a**, Schematic of experimental setup in which a 7–9-dpf larval zebrafish is embedded in agarose and imaged in a custom-built lightsheet microscope using a lateral excitation beam. **b**, Left, expression pattern of the *Tg(gad1b:Gal4)* line over a brain reference. Center, example view from an imaging experiment. The blue box indicates the area imaged in the light sheet experiments. r1π ROIs are highlighted in pink over the shades of all ROIs from the experiment. Right, example traces from one experiment. **c**, Frontal, horizontal and sagittal projections of the r1π neurons from all experiments registered in a common anatomical space (pink), visualized together with all of the ROIs extracted from the same experiments (gray). The ROIs are shown on the mapzebrain atlas (https://mapzebrain.org). **d**, Circular propagation of activity. Intensity of fluorescence for all ROIs in one fish in the course of a clockwise (cw, top) and anticlockwise (acw, bottom) propagation event. The arrow shows the direction of activity propagation. **e**, Trajectory in the first two PCs of the phase space of the network, color coded by time. **f**, Left, projection over the first two (rotated) PCs in time of all of the r1π neurons, color coded by angle around the circle (for rPC calculation, see Methods and Supplementary Fig. 1). Right, anatomical distribution of the same neurons, color coded by angle in PC space. **g**, Projection of ROIs pooled from all neurons in the registered rPC space, color coded by angle in rPC space (left) and their anatomical distribution (right). **h**, Traces of r1π neurons, sorted by angle in PC space for the neurons and phase of the network (green line). ΔF/F, relative change in fluoresence; Cau., caudal; Dors., dorsal; mhb, midbrain/hindbrain boundary; OT, optic tectum; post., posterior; rh, rhombomere; Ros. rostral; *Z* sc., *Z* score.

**Extended Data Fig. 1:**
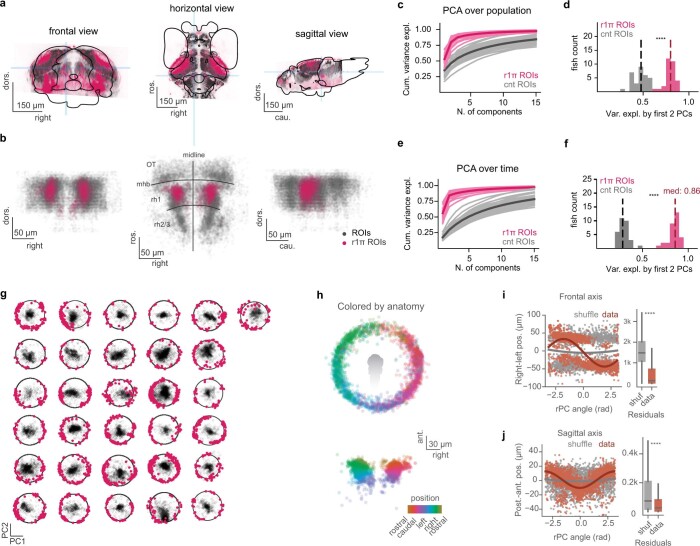
Anatomical location of r1π neurons and PCA decomposition of their activity. **a**, Frontal, horizontal and sagittal projection of the expression pattern of the gad1b:Gal4 used in the imaging experiments from one fish. In the gray background, the expression pattern of elavl3:H2B-mCherry, on a second channel in the same fish. The blue shades indicate the slices of the stack that were averaged to obtain the views, and are centered on the location of the imaged GABAergic nuclei in the aHB. **b**, The same views for the r1π neurons in the imaging experiments registered in a common anatomical space (pink), visualized together with all the ROIs extracted from the same experiments (gray). OT: optic tectum, mhb: midbrain/hindbrain boundary, rh: rhombomere. **c**, Cumulative relative variance explained by the first 15 components of PCA decomposition over population for r1π neurons (purple) and a population of randomly drawn neurons from the same imaging experiments matching in number the r1π neurons (gray). Light lines: individual fish, dark thick line: population average. **d**, Variance explained by the first two PC components in the plot in (a), compared between r1π and control neurons (Wilcoxon test, p < 0.0001). **e**, Cumulative variance explained by the first 15 components of PCA decomposition over the time dimension, legend as in (a). **f**, Variance explained by the first two PC components in the plot in (c), compared between r1π and control neurons (Wilcoxon test, p < 0.0001). **g**, Projections over first two principal components calculated over the r1π neurons (in pink) of all neurons (in gray) for each fish, for all fish in the dataset. **h**, Projection of ROIs pooled from all neurons in the registered rPC space, color coded by their angle in the anatomical space. **i**, Fit of a sinusoidal wave to the anatomical position on the right-left position, as a function of the ROI phase in rPC space (left), and distribution of square distances of ROIs from the fit (right) (p < 0.001, Mann-Whitney U test, n = 1330 ROIs from 31 fish). The fit was computed over 50% of the ROIs, and the residuals calculation over the left-out 50%. The box plot spans the range between the first and the third quartile, and the median is marked; the whiskers extend to the 5th and the 95th percentiles of the distribution. **j**, As in (i), for the antero-posterior axis (p < 0.001, Mann-Whitney U test, n = 1330 ROIs from 31 fish).

We performed volumetric lightsheet imaging (Fig. 1a) in 7–9-days postfertilization (dpf) zebrafish larvae expressing GCaMP6s in GABAergic neurons in the aHB (Fig. 1b and Extended Data Fig. 1a). Larvae were head restrained but free to move their tail, and were imaged either in darkness or while presented with a visual stimulus in either closed or open loop (see Visual stimuli and experimental groups in the Methods). We observed a population of 50–100 neurons (median = 74, quartile 1 (*Q*_1_) = 48, *Q*_3_ = 115, *n* = 31 fish) with a sustained bump of activity propagating either clockwise or anticlockwise across the network in a horizontal plane (Fig. 1c,d and Supplementary Video 1). These GABAergic neurons were located in rhombomere 1 consistently across fish (Fig. 1c and Extended Data Fig. 1b; see Methods for a description of the selection process of this group of neurons). To further characterize the dynamics of the network, we performed principal component analysis and observed that the first two principal components (PCs) captured over 80% of the variance (median = 0.800, *Q*_1_ = 0.770, *Q*_3_ = 0.836, *n* = 31 fish) (Extended Data Fig. 1c,d). Moreover, the trajectory in the phase space defined by the first two PCs was constrained to a circle over the whole duration of the experiment which lasted tens of minutes (Fig. 1e). We named these cells r1π neurons because of their location in rhombomere 1, the fact that they have an anticorrelated partner at a π angle on the PC space and on the basis of their morphological features described later in this paper.

**Extended Data Fig. 2:**
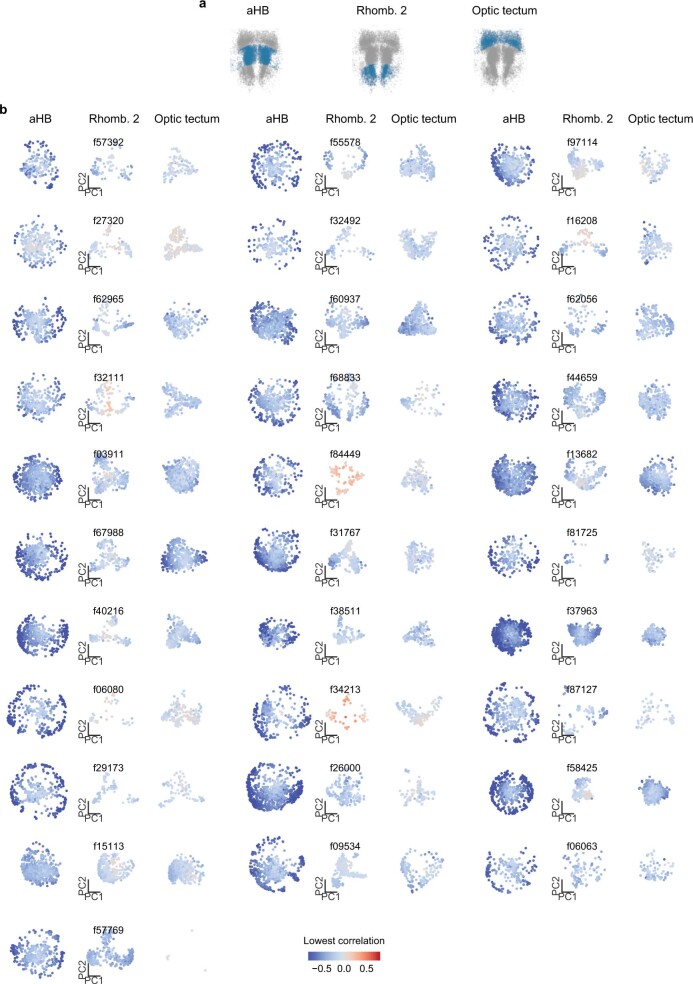
Circular distribution of PC projections is a feature of aHB. **a**, ROIs coarsely grouped into anatomical regions using manually defined borders for optic tectum, anterior hindbrain and rhombomere 2. **b**, For every fish, principal components over time were computed over all neurons of each region, without any selection. Then, all ROIs were projected over the first two components. ROI are color coded by the maximum anticorrelation with any other cell in the dataset, the criterion that was used to define r1π neurons throughout the paper.

To visualize how the activity and anatomical location of r1π neurons were related, we projected the activity of each neuron onto a two-dimensional subspace by performing a different PC projection, now over the time axis. When projected over the first two PCs (variance explained, median = 0.858, *Q*_1_ = 0.827, *Q*_3_ = 0.868, *n* = 31 fish) (Extended Data Fig. 1e,f), r1π neurons were organized in a circle, with the angle around the circle, *α*, correlating with the neuron’s anatomical location (Fisher–Lee circular correlation *ρ*_t_, median = 0.549, *Q*_1_ = 0.298, *Q*_3_ = 0.696, *n* = 31 fish) (Fig. 1f,g, Extended Data Fig. 1g–j and Supplementary Figs. 1 and 2). This matches the observation from the raw data of a bump of activity propagating across the network: the circular dynamics we observe in phase space corresponds to the activity propagation across an anatomical circle of neurons. The activity of neurons from neighboring areas (optic tectum and rhombomere 2) did not show the same distribution when projected over the first PCs, suggesting that the circular distribution seen with r1π neurons is a specific feature of aHB neurons (Extended Data Fig. 2).

**Extended Data Fig. 3:**
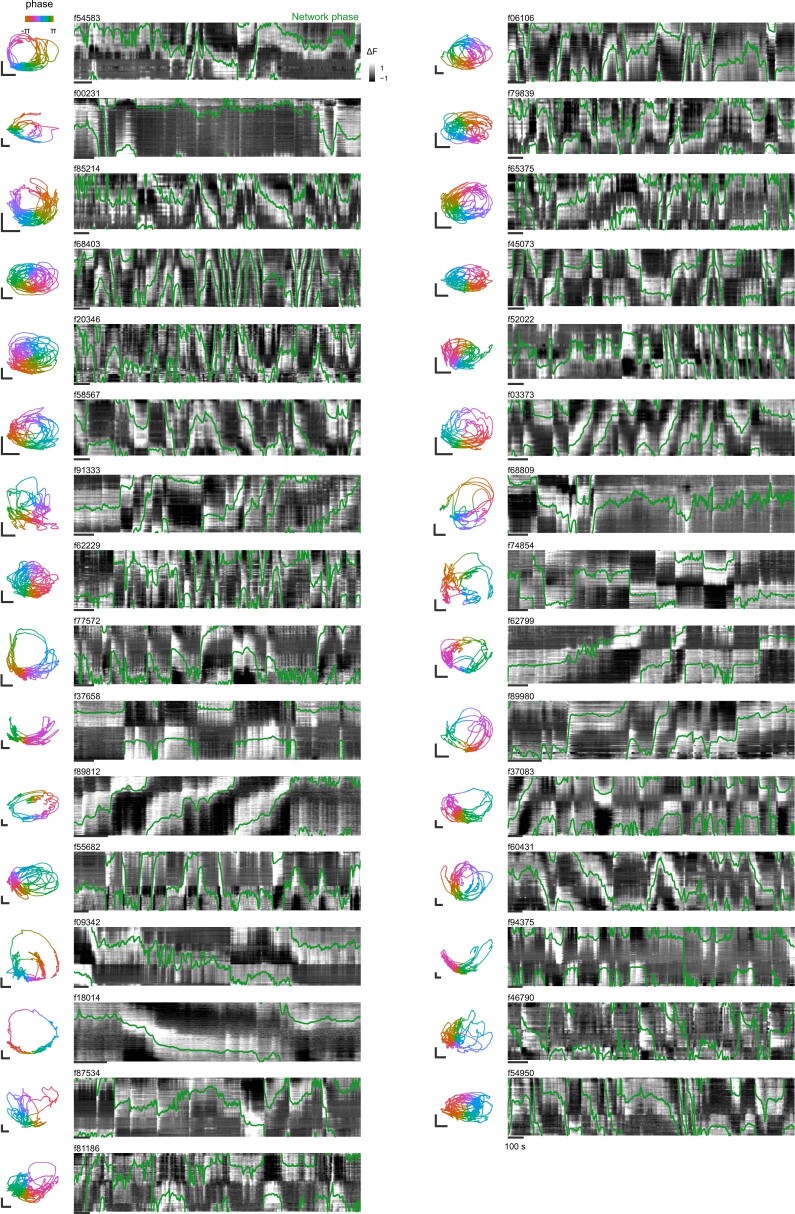
Summary plot for all fish. Raw data showing for each fish of the dataset the trajectory of the network in PC space over the entire duration of the experiment color coded by network phase (panel on the left), and the raw traces of r1π neurons sorted by neuron angle θ and network phase in green. The scale bar in the phase space has length of 5, and the bar below the traces indicates 100 s.

To visualize evolution of the network activity over time, the traces of neurons were sorted by their angle *α* (Fig. 1h and Extended Data Fig. 3). This visualization showed how the phase marks the position of the bump peak as it translates across the network.

**Fig. 2:**
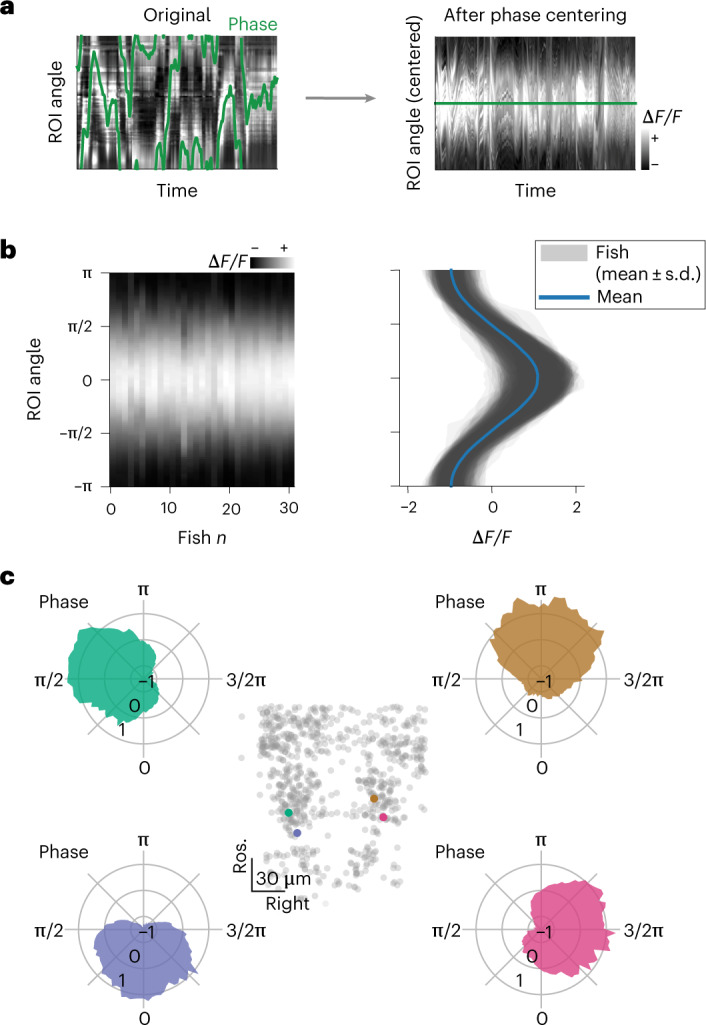
Network activity profile. **a**, Phase-zeroing process: for every time point, a circular permutation of the (interpolated) activity matrix was computed so that the peak of activation, mapped by the phase (left), was always in the center of the matrix. Right, the matrix of traces after the centering. **b**, Profile of the activity bump. Left, matrix showing the average activation profile for all fish in the dataset (*n* = 31 fish). Right, mean ± s.d. over time for each fish (shaded areas) and population average (blue). **c**, Polar plots showing tuning curves for the activation of individual neurons as a function of network phase from one fish. Each panel shows the curve for a neuron, color coded by their angle in rPC space *θ*. The anatomical locations of the four neurons are shown in the central inset.

To describe the position of the bump of activity within the network at any instance in time, we defined an instantaneous network phase *φ*(*t*) as the angle of the vector average over neurons, weighted by their activity at time *t*, over their first two rPC projections (Supplementary Fig. 3a and Supplementary Video 2). This network phase *φ*(*t*) describes the angle along the circular trajectory in the network phase space (Fig. 2a).

We anchored *φ* by setting it to be zero when the posterior part of the ring was active and to increase with clockwise rotations of activity in the horizontal plane (Methods and Supplementary Figs. 1 and 3a).

To further characterize how individual neurons contributed to the network activity, we computed the average network activity over time (Methods, Fig. 2a and Supplementary Fig. 4). The network activity profile approximated a sine wave, with a full width at half maximum of π radians (rad) (mean ± s.d. = 2.91 ± 0.115, *n* = 31 fish) over the circle of neurons (Fig. 2b and Supplementary Fig. 4b). The tuning curves of individual neurons over the network phase had a sinusoidal shape, with full width at half maximum of approximately π rad (Fig. 2c).

### The r1π network integrates heading direction

**Fig. 3:**
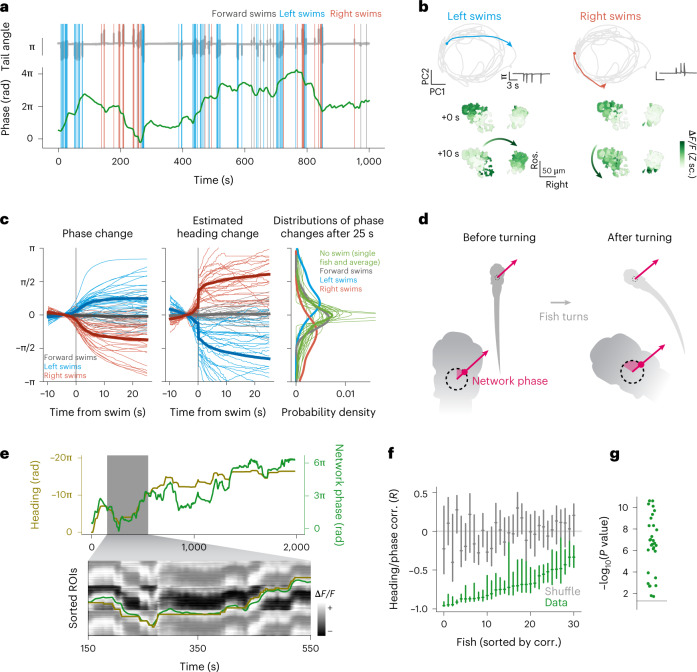
The aHB network tracks estimated heading angle over many minutes. **a**, Network phase and motor activity. Top, tail angle over time (gray). Colored vertical lines mark the occurrence and direction of swims. Bottom, unwrapped network phase (green) over time. **b**, Network trajectory during sequences of left and right swims. Top left, trajectory in phase space during a sequence of left swims (see tail angle in the inset on the right of each plot). Bottom left, state of activation of the network before and after the sequence. Right, a sequence of right swims. **c**, Left, swim-triggered average change in network phase for all fish (thin lines, *n* = 31 fish) and their overall average (thick lines). Center, swim-triggered change in estimated heading. Right, histograms of the accumulated phase change 25 s after a swim (blue, red and gray lines), together with fish-wise histograms of the spontaneous drift of the network phase in 25 s when no swim occurred (thin green lines, individual fish; thick line, average). **d**, Schematic to show how the network phase changes during a turn, and keeps pointing in the same direction in allocentric coordinates. **e**, Top, network phase (green) and estimated fish heading (gold) for the entire duration of an experiment. Note the axes are different and have opposite signs. The inset shows the same traces, overlaid on the traces from the r1π neurons, tiled to match the phase unwrapping. **f**, Correlation (corr.) of heading and network phase for all fish in 5-min chunks, compared with a shuffle of the same data. Bars report median and *Q*_1_ and *Q*_3_ for the data (green) and shuffle (gray). **g**, Distribution of *P* values for the comparison of correlation of phase and heading in the data and shuffle for each fish (Wilcoxon rank-sum test, *P* < 0.01 for *n* = 31 fish).

Next, we investigated what was driving changes in the phase of the network. The phase was stable in periods when the fish was not moving, and it changed the most during sequences of left or right swims (Fig. 3a).

**Extended Data Fig. 4:**
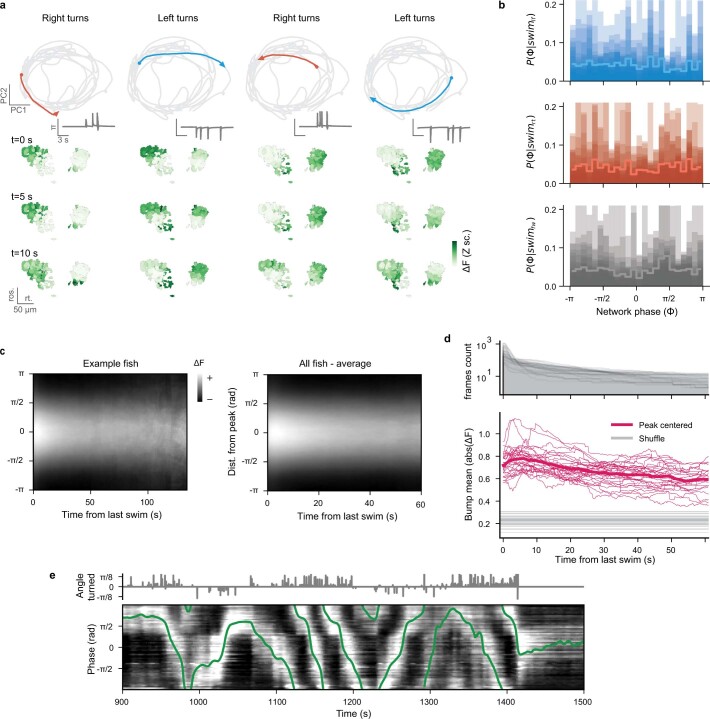
Phase dynamics during directional swims. **a**, Network trajectory during sequences of left and right swims (same as Fig. 3b, with more examples.). Top, trajectory in phase space during a sequence of left swims (see tail angle in the insert). Bottom, state of activation of the network before and after the sequence. The four columns show four sequences of left and right turning, with the network starting at different phases. **b**, Probability of network phase given that a forward, left, or right swim occurred (shaded areas: individual fish; line: population average, n = 31 fish). The distribution is flat, suggesting that the network phase is not instructive with respect to the direction of swimming. **c**, Average activation profile, interpolated and phase-centered as shown in S8, during stretches of time where no swim occurred, for a single fish (top) and averaged across all fish (bottom). **d**, (bottom), Quantification of the bump amplitude shown in d, for every fish (thin pink lines) and their average (thick pink line), compared with the mean values obtained by averaging a randomly drawn distribution of non phase-centered bump profiles (thin gray lines) and their average (thick gray line). Above, number of frames used to compute average profile below for every fish. **e**, Example of clockwise and counter-clockwise shifts traversing multiple times the entire network during sequences of repeated directional swims.

Furthermore, sequences of left or right turns were accompanied by respective clockwise or anticlockwise rotations of the network phase (activity), irrespective of the starting phase position (Fig. 3b and Extended Data Fig. 4a).

To quantify this relationship, we computed the swim-triggered change in phase and noticed that it was consistently increasing or decreasing after left and right swims (Fig. 3c); therefore, left swims (anticlockwise rotations of the fish) would produce clockwise rotations in the network, and right swims (clockwise rotations of the fish) would produce anticlockwise rotations in the network. Forward swims did not produce any consistent change in the network phase. Importantly, when the fish was not actively swimming, the bump persisted over several tens of seconds over the same part of the network (Fig. 3c, right, and Extended Data Fig. 4c,d).

Importantly, we observed that the probability for the network phase to be in any state between −π and π when a swim occurred was not different for left, right or forward swims (Extended Data Fig. 4b). This indicates that the absolute or instantaneous network phase does not correlate with a specific swim direction and suggests that these neurons are not coding for left or right turns, but rather for the heading direction of the animal.

To further investigate this hypothesis, we extended our analysis of swim-triggered changes in network phase (Fig. 3c, left). The magnitude of change in angle elicited by a single swim after 10 s was approximately π/4 (median = 0.828 rad, *Q*_1_ = 0.492 rad, *Q*_3_ = 1.28 rad, *n* = 31 fish), which is comparable in size to the angle turned by a swim performed by a freely swimming fish (Fig. 3c, middle, and Extended Data Fig. 4)^18^. Moreover, continuous turning in one direction resulted in several rotations around the network (Extended Data Fig. 4e). This shows that the network could function as a heading direction integrator, shifting the position of its activity with every turn and keeping track of the heading direction of the animal, as illustrated in Fig. 3d.

**Extended Data Fig. 5:**
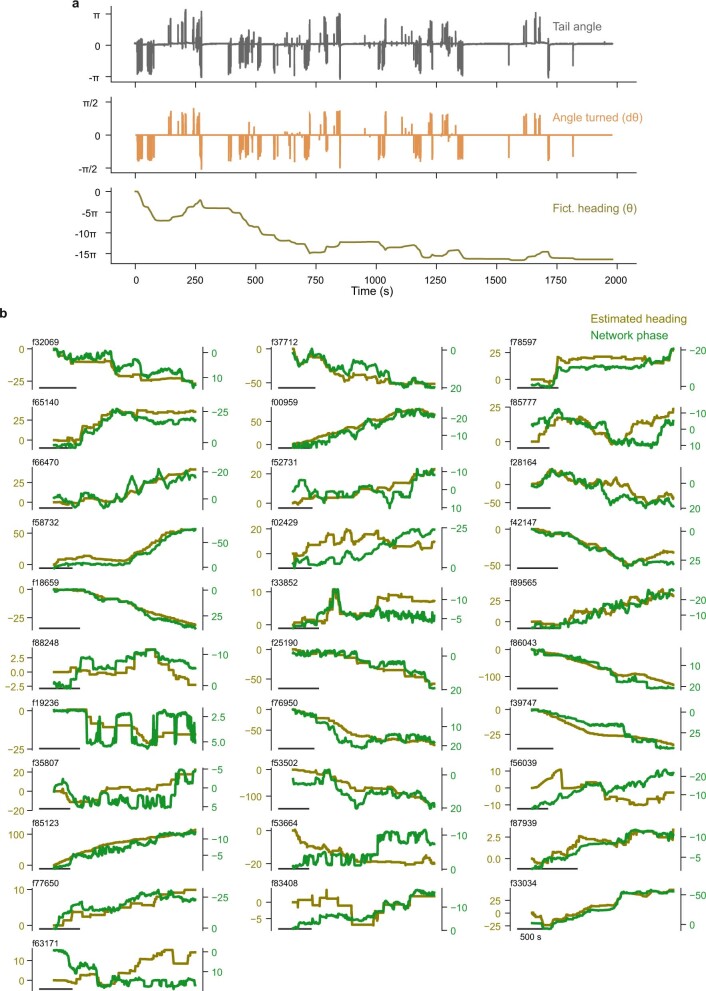
Phase predicts fictive heading. **a**, Calculation of the fish heading estimate. (top) Raw tail trace. (middle) Laterality index for each swim. (bottom) Cumulative sum of laterality indexes of swims. **b**, Raw estimated heading (green line) and phase (golden line) for all fish in the dataset.

To understand the degree at which the network could produce an estimate of heading direction over time, we reconstructed a fictive heading direction for the head-embedded fish, integrating the angle turned by each swim over time (Methods and Extended Data Fig. 5a). The reconstructed heading direction and the network phase were anticorrelated over a period of minutes (Fig. 3e).

Although some errors accumulated over time, the network phase around each time point could be used to estimate the current heading direction and these two variables were significantly anticorrelated (median correlation = −0.723, *Q*_1_ = −0.863, *Q*_3_ = −0.564, *n* = 31, *P* < 0.01 compared with a control shuffle for each fish) (Fig. 3e–g and Extended Data Fig. 5).

### The r1π network is not affected by visual input

Next, we asked whether sensory inputs are required for the observed heading direction integration. Because the zebrafish were head restrained (Fig. 1a), we ascertained that vestibular sensory inputs were not required, although they are known to contribute to the mammalian heading direction system^19^.

**Fig. 4:**
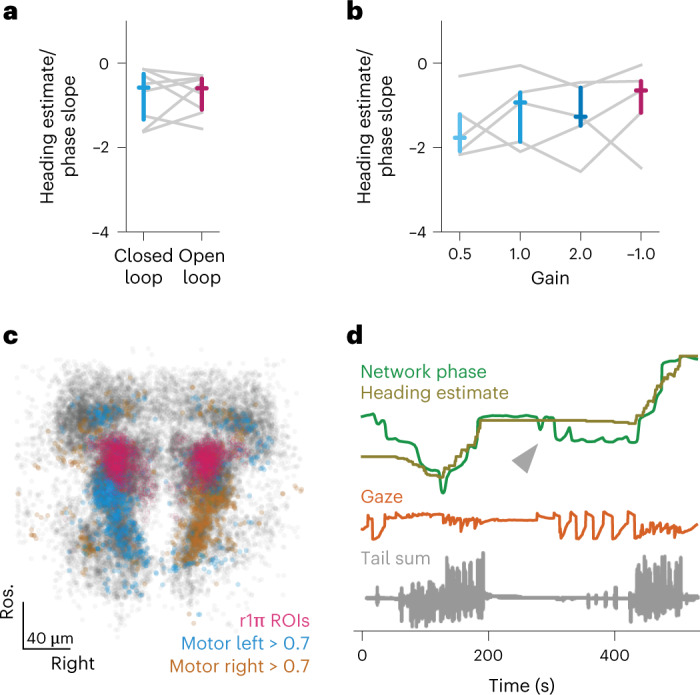
Tracking of heading angle does not depend on visual feedback but may incorporate eye gaze direction. **a**, Slope of the regression between estimated heading and network phase in closed-loop and open-loop epochs (closed-loop median = −0.579, *Q*_1_ = −1.34, *Q*_3_ = −0.257 versus open-loop median = −0.595, *Q*_1_ = −1.1, *Q*_3_ = −0.37; *P* = 1.00, two-sided Wilcoxon signed-rank test, *n* = 8 fish). Colored marks indicate median and the *Q*_1_–*Q*_3_ range. **b**, Slope of the regression between heading and network phase in different gain conditions; comparison was not significant between any of the conditions (two-sided uncorrected Wilcoxon signed-rank test, *n* = 5 fish). Colored marks indicate median and the *Q*_1_–*Q*_3_ range. **c**, Distribution of directional swim-related ROIs (*n* = 31 fish). All ROIs from all fish are shown in gray, r1π neurons are shown in pink, neurons showing high correlation (more than 0.7) with left and right turning are shown in blue and orange, respectively. **d**, Example of a recording showing the contribution of gaze direction to the network signal when the fish is not swimming. Network phase (green) represented with the (inverted) heading direction (golden) and gaze direction (orange). The arrow highlights a period of no swimming and large saccades.

**Extended Data Fig. 6:**
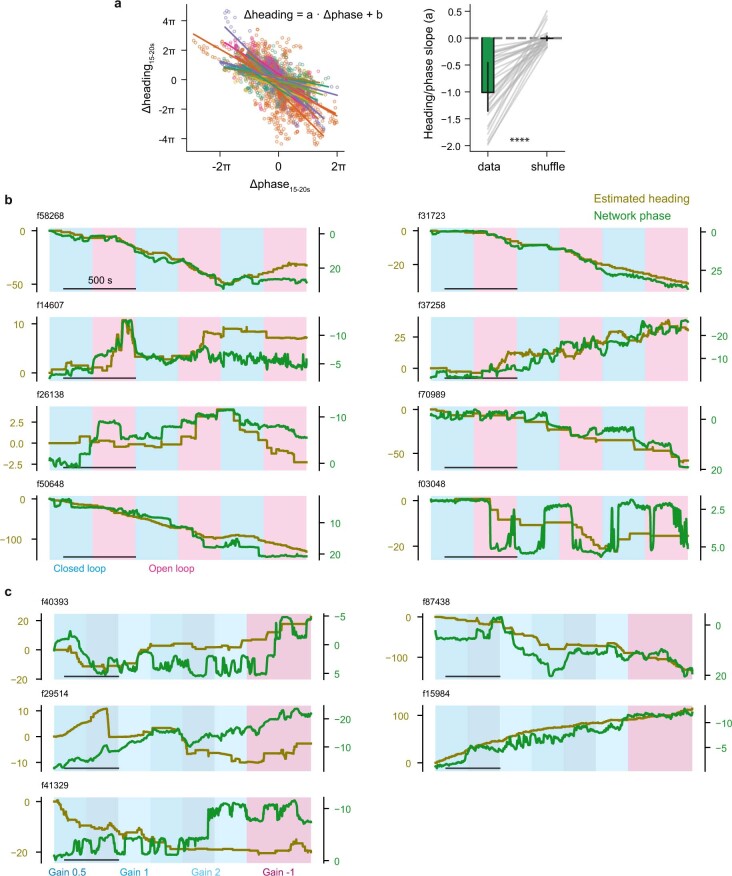
Visual feedback is not required for the turning integration. **a**, To quantify the effect of visual feedback on the network activity, we performed a linear regression between the amount of shift in the network phase and in the estimated heading, between ~15 s after each swim (see Methods). Left, scatter plot of heading change vs phase change after every swim (individual points, color coded by fish), and the linear fit for every fish. Right, comparison between the slopes in the data vs. a shuffle over swim identity (data: median = −1.01, Q1 = −1.36, Q3 = −0.457; shuffle: median = −0.00647, Q1 = −0.0452, Q3 = 0.0343; p < 0.0001, Wilcoxon test). **b**, Heading and network phase with the experiment condition for the closed-loop open-loop experiments (pink shading: open-loop epochs; blueshading: closed loop epochs; scale bar: 500 s). **c**, Heading and network phase with the experiment condition for the gain modulation experiments (dark blue: gain 0.5, light blue: gain 2; blue: gain 1; red: gain −1).

In our experiments, we tested a variety of visual stimuli conditions (Supplementary Fig. 6) and observed the integration of heading direction in both closed-loop (with visual reafference coherent with the direction of the swim) and in open-loop experiments (without visual reafference) (Fig. 4a and Extended Data Fig. 6a,b), indicating that visual feedback is not required for a stable heading direction representation. Furthermore, we performed closed-loop experiments with a range of gain conditions that provided different amounts of visual feedback. In these experiments, we observed no relationship between the representation of heading direction and the experimental gain (Fig. 4b and Extended Data Fig. 6c). This shows that visual feedback is not required for this representation, and suggests that efference copies are the main driver.

**Extended Data Fig. 7:**
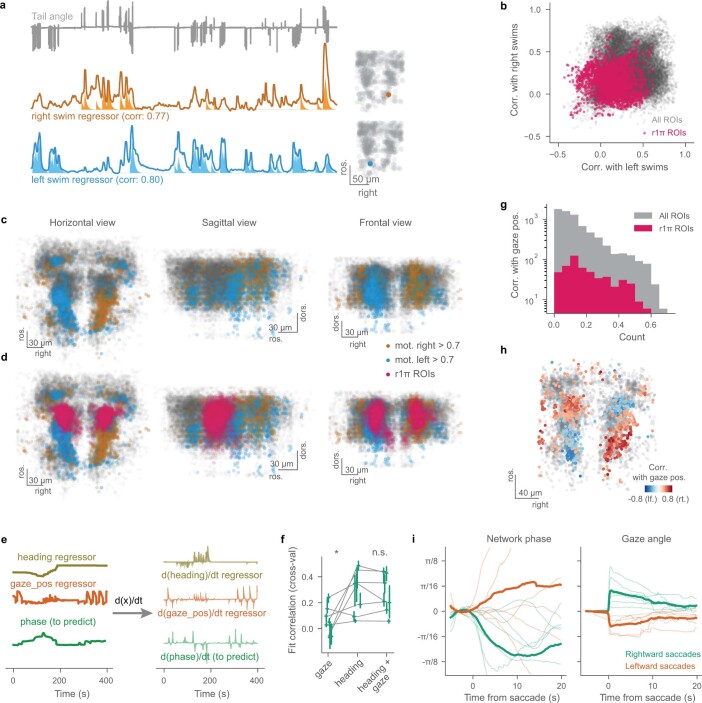
Motor related signals in the aHB. **a**, Example traces for right and left swims-selective neurons together with the fish tail trace (left), and their anatomical location (right), shown on top of a scatter plot of all ROIs from the same fish. The regressors are shaded below the traces, and the correlations of the traces with the regressors are reported in the plot. **b**, Correlations of all ROIs and r1π ROIs with left and right swims regressors. **c**, Horizontal, sagittal and frontal view showing all ROIs that have a correlation > 0.7 with a regressor for swims in one direction and < 0.7 with the regressor for swims to the opposite side (blue: left swims; golden: right swims). **d**, Same plot as in c, showing also all the r1π neurons. **e**, Illustration of the procedure for fitting the phase. We performed either linear regression or multiple linear regression to fit the network phase from heading and/or gaze. For the fit we used the time derivatives of those quantities (right). See Methods. **f**, Correlation of the reconstructed test phase and the actual phase when the prediction was performed using only the gaze information, using only the heading information, or a combination of both. Gaze: median = 0.0507, Q1 = −0.0155, Q3 = 0.125; heading: median = 0.348, Q1 = 0.139, Q3 = 0.404; gaze + heading: median = 0.209, Q1 = 0.173, Q3 = 0.391. Comparisons: Wilcoxon test, n = 7 fish. **g**, Histogram of the correlation of r1π neurons with a gaze position regressor, compared with the distribution obtained from all ROIs. **h**, Anatomical view (horizontal projection) of the correlation values of neurons with a gaze position regressor. **i**, Saccade-triggered phase changes in the network (left), and gaze deflections (right) for rightward (green) and leftward (orange) saccades.

Interestingly, the activity of left and right GABAergic clusters in rhombomeres 2 and 3, immediately caudal to the r1π neurons, show a remarkable degree of correlation with leftward and rightward swims, respectively (Fig. 4c and Extended Data Fig. 7a–d). These neurons might provide the motor efference input to the r1π network, although further experiments would be required to prove their involvement in the network.

### The r1π network is modulated by eye movements

Subpopulations of cells in the aHB are known to represent eye-related variables, such as eye position and saccade timing^20,21^. Therefore, we investigated whether eye motion could also modulate the network phase.

To this end, we freed the eyes of the larvae in a subset of experiments and tracked their motion together with that of their tails. In periods of no swimming, eye motion could explain some low-amplitude modulation in the network phase (Fig. 4d and Extended Data Fig. 7g–i); however, when swimming did occur, eye motion alone performed poorly compared with heading direction (Extended Data Fig. 7f).

Interestingly, the sign of the modulation was consistent with the changes in heading, with leftward saccades increasing the network phase as do leftward swims, and rightward saccades decreasing it as do rightward swims.

### aHB neurons arborize in the dIPN

**Fig. 5:**
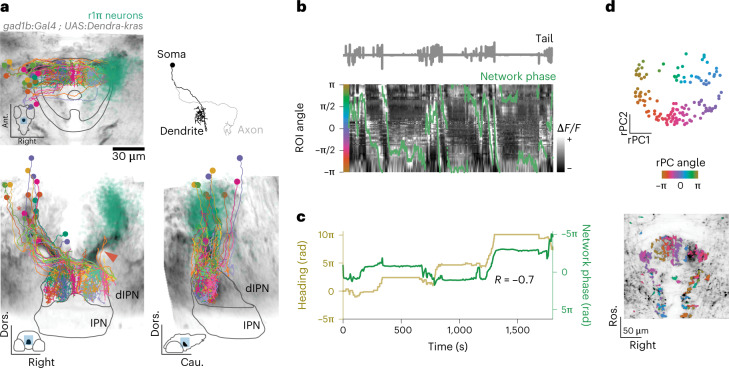
r1π neurons form reciprocal connections in the dIPN. **a**, Anatomical projections of a stack from the *Tg(gad1b:Gal4);Tg(UAS:Dendra-kras)* line used in the experiments. The lines mark the IPN and dIPN boundaries, and the insets show the position in the brain of the IPN mask and the orientation. The red arrowhead highlights the tract of fibers that extend from the aHB to the IPN. The r1π neurons from the imaging experiments are shown in the same coordinate space in green on the right, together with the morphology of all neurons reconstructed in the EM on the left. Top right, morphology of the r1π neuron marked by the asterisk in the bottom-left projection. **b**, Traces of ROIs in the dIPN showing r1π-like dynamics, sorted by angle in PC space, and phase of the network (green line). The tail trace is shown in gray above. **c**, Estimated heading direction (gold) and network phase (green) are highly correlated. **d**, Top, projection over the first two rPCs in time of all of the ROIs showing r1π-like activity, color coded by angle around the circle. Bottom, anatomical distribution of the same neurons, color coded by angle in rPC space. The anatomy of the recorded plane is shown in the background. Dors., dorsal.

**Extended Data Fig. 8:**
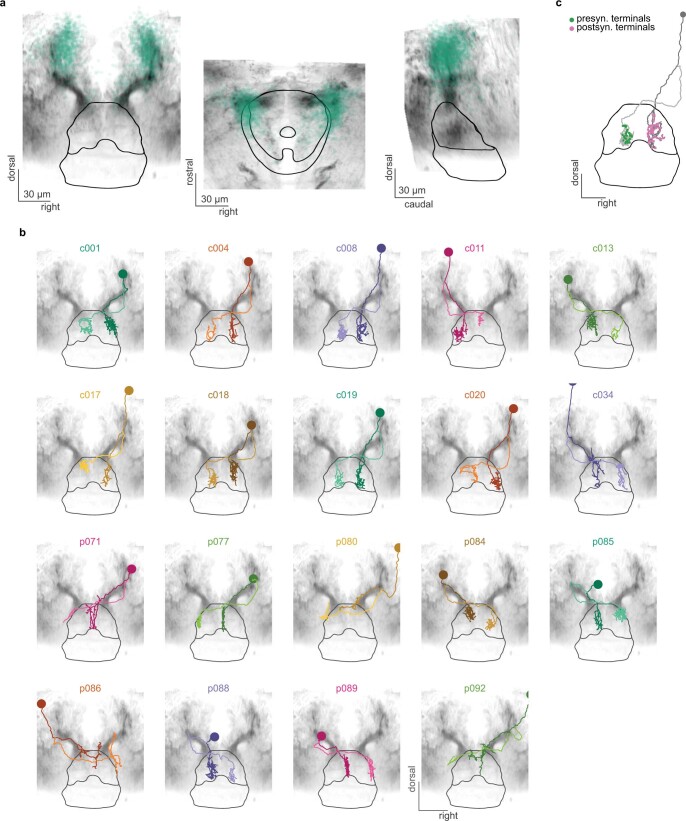
r1π neurons form reciprocal connections in the IPN. **a**, Frontal, horizontal and sagittal view from gad1b:Gal4, UAS:Dendra-kras stack in the region around the IPN. A scatter plot representing the position of all r1π neurons from the functional dataset mapped to the IPN reference space is shown in green. **b**, Individual plots of EM reconstructed neurons. Frontal view for all neurons presented in Fig. 5. **c**, Localization of presynaptic terminals and postsynaptic terminals from one cell in the dataset, as reconstructed by annotators. All postsynaptic nodes are on the ipsilateral shaft (the dendrite), while all presynaptic ones are on the contralateral shaft (the axon).

We proceeded to investigate the anatomy of neurons in the aHB. Anatomical stacks of our GABAergic line show a prominent, bilaterally paired tract of fibers that extend ventromedially from the GABAergic nuclei of rhombomere 1 toward the dIPN (Fig. 5a, red arrow, and Extended Data Fig. 8a).

To reconstruct individual neurons at high resolution, we traced neurons and their projections in a serial block-face EM (SBEM) dataset. We identified a neuron class in which the soma, located in the aHB, extended a single projection that bifurcated into a dendrite and axon, both of which terminated in the dIPN (Fig. 5a, Supplementary Video 3, Extended Data Fig. 8b and Supplementary Fig. 7).

The small dendritic tree covered a localized compartment in the ipsilateral IPN, whereas the axon projected contralaterally with minimal branching which occurred only in the terminal sections (Extended Data Fig. 8b,c).

**Extended Data Fig. 9:**
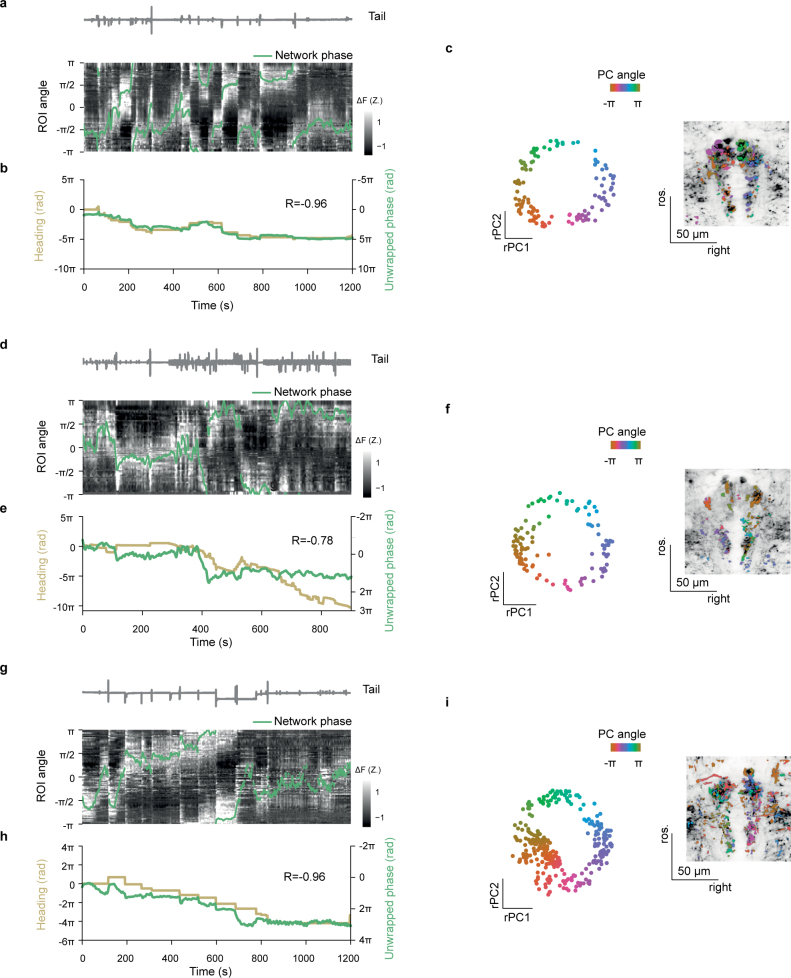
r1π neuron-like activity in the dIPN. Three example datasets are shown. **a**, **d**, **g**, Traces of ROIs in the dIPN showing r1π-like dynamics, sorted by angle in PC space, and phase of the network (green line). The tail trace is shown in gray on top. **b**, **e**, **h**, Estimated heading direction (gold) and the unwrapped network phase (green). **c**, **f**, **i**, left, projection over the first 2 PCs in time of all the ROIs showing r1π-like activity, color coded by angle around the circle. right, anatomical distribution of the same ROIs, color coded by angle in PC space. The anatomy of the recorded plane is shown in the background.

To confirm that r1π neurons project into the dIPN, we imaged the same GABAergic line under a two-photon microscope to investigate neuropil activity. Performing the same analysis as for the r1π neurons presented in Fig. 1, we discovered a set of regions of interest (ROIs) that were mostly restricted to the dIPN, showed stable circular dynamics and displayed the same relationship to heading direction (Fig. 5b–d and Extended Data Fig. 9).

### aHB projections map circular aHB activity to linear dIPN activity

It has been suggested that heading direction systems are neuronal implementations of ring attractor networks, where excitatory activity between neighboring cells is stabilized and localized by long-range inhibitory connections. Therefore, we wanted to investigate whether there is any evidence that the morphology and projections of the GABAergic r1π neurons could implement such a structure.

**Fig. 6:**
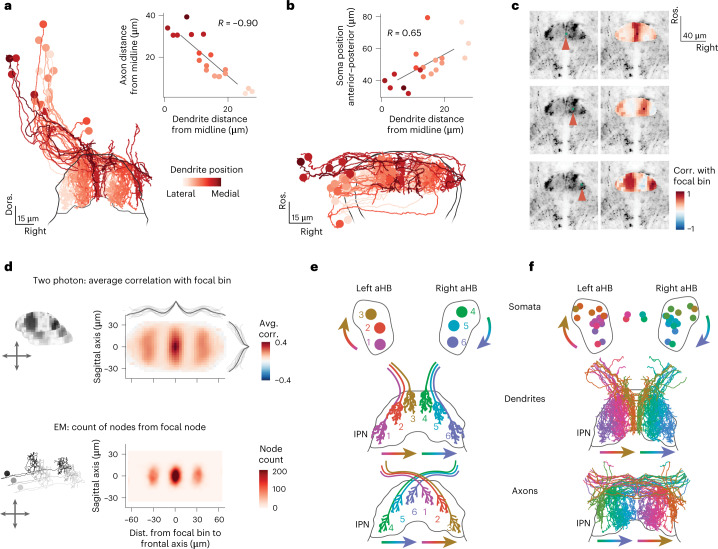
Organization of dendrites and axons from the aHB within the dIPN. **a**, Frontal view of the IPN-projecting aHB neurons, color coded by position of the dendrite on the coronal axis (lateral–medial). Inset, scatterplot of the distance from the midline of dendrite and axon for each neuron (*R* = −0.90, *n* = 19). **b**, Horizontal view of the IPN-projecting aHB neurons, color coded by position of the dendrite on the coronal axis (lateral–medial). Inset, scatterplot of the distance from the midline of dendrite and position of the soma of the anteroposterior axis for each neuron (*R* = 0.65, *n* = 19). **c**, Anatomy of *Tg(gad1b:Gal4);Tg(UAS:GCaMP6s)* from a two-photon experiment (left) with a focal bin highlighted by the red arrowhead, and maps of correlations (corr.) of bins with the focal bin (right). Each row corresponds to a different focal point. **d**, Top, average correlation of bins at different distances around a focal bin in the two-photon data. The lines on the side show the means of individual fish across each axis (thin lines) and the population average (thick line, *n* = 11). Bottom, counts of nodes around a focal node in the EM data. **e**, Schematics of the organization of aHB neuron somata (top) and their dendritic (middle) and axonal (bottom) projections in the IPN. **f**, Reconstructed EM neurons mirrored on both sides and color coded on the basis of the position of the dendrite, following the schema in **e**. Avg., average; Dist., distance.

To this end, we returned to the SBEM reconstructions. The projections of different neurons occupy different locations in the mediolateral axis and appear to cover the whole dIPN (Fig. 6a, Supplementary Video 3 and Extended Data Fig. 8b).

**Extended Data Fig. 10:**
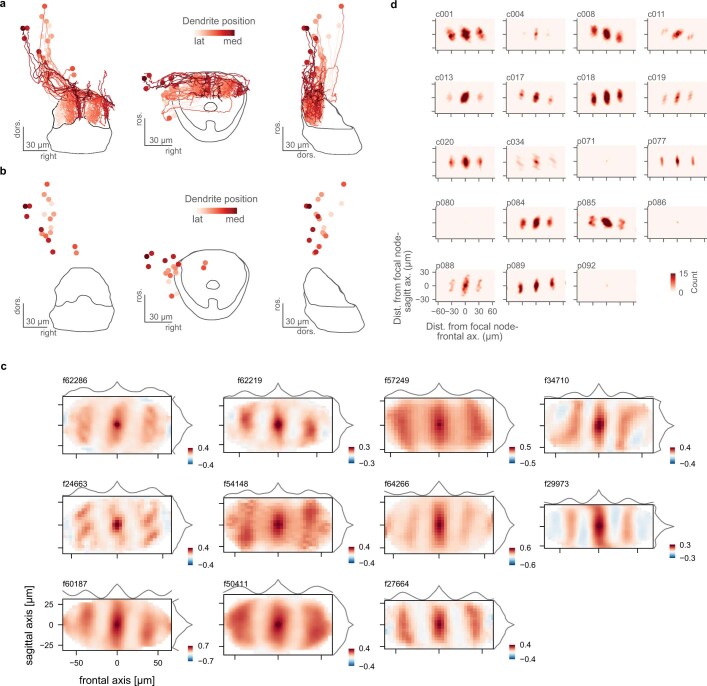
The organization of IPN projecting neurons of the aHB. **a**, Complete frontal, horizontal and side view for the data presented in Fig. 6a. **b**, The same views, now showing only the soma locations. **c**, 2D auto-correlation of the activity in the dIPN for all fish in the dataset. Each matrix shows the mean correlation between a focal bin and the other bins at different distances in a two-photon plane for each fish. The lines on the side show the means across each axis. **d**, Plots of node distances for each reconstructed neuron; those data were summed to obtain the panel in Fig. 6d (bottom).

Furthermore, the distance of the dendrite from the midline was anticorrelated with the distance of the axon from the midline (*r* = −0.9, *n* = 19 neurons), meaning that a neuron with a lateral dendrite would extend a medial axon and vice versa (Fig. 6a and Extended Data Fig. 10a). In addition, given the anatomical organization of the activity in the aHB, we expected a correlation between the anteroposterior position of a cell’s soma and the distance of its dendrites from the midline. Indeed, this is what we observed (*r* = −0.65, *n* = 19 neurons) (Fig. 6b and Extended Data Fig. 10b).

Such an organization would predict that, in the activity recorded from the dIPN, pixels that are the most correlated with each other are at a fixed distance on the mediolateral axis, because their signal originates from the dendrites and axons of the same neurons. Indeed, we observed this pattern when examining data from a single fish (Fig. 6c) and across all fish (Fig. 6d, top, and Extended Data Fig. 10c).

The distance of the side lobes observed in the functional correlation was associated with the distance of each neuron’s axon from its dendrite (Fig. 6d, bottom, and Extended Data Fig. 10d).

These observations suggest that a circular functional structure in the aHB, corresponding to angles from −π to π, is coupled to a linear structure in the dIPN (Fig. 6e). The axons of neurons whose somata are at opposite sides of the circular organization of the aHB target their respective opposing dendrites (Fig. 6f). In this way, a neuron is ideally placed to inhibit its corresponding out-of-phase neuron. This projection pattern could stabilize and localize the heading direction activity we observed in the aHB network by providing long-range inhibition.

## Discussion

In this study, we describe a network of GABAergic neurons in rhombomere 1 of the larval zebrafish hindbrain that encodes the heading direction of the fish. Because this representation persists even in the absence of external landmarks and salient sensory stimuli, it is likely generated by the integration of efference copies. We show that motor-related activity exists nearby that may be serving this purpose, in agreement with previous reports^22,23^. This observation confirms the existence of an internal model of turning in the zebrafish brain.

Remarkably, heading direction is represented by a bump of activity that propagates clockwise and anticlockwise in the horizontal plane with leftward and rightward movements of the fish. To our knowledge, this is the first evidence of an anatomical organization for the heading direction system in a vertebrate and suggests the existence of simple topographical principles in the wiring of the network. Indeed, our EM reconstructions suggest that heading direction neurons of the aHB connect to each other in a precise way, with neurons whose functional activation is in complete antiphase making reciprocal connections in the dIPN. On the basis of their location and their consistent antiphase projection pattern, we named these cells r1π neurons.

In mammals, the GABAergic^24,25^ DTN is considered to be one of the earliest subcortical structures within the heading direction pathway, and tracing studies have identified reciprocal connections between the DTN and the IPN^26–28^. The heading direction neurons observed in the DTN are broadly tuned^10^, similarly to the neurons we report in the fish aHB. Moreover, tegmental afferents to the mammalian (rostral) IPN form highly compartmentalized arborizations^29,30^, similar to what we observe in the reconstructions of aHB fibers.

Theoretical studies^16,17,31,32^ proposed the notion of ring attractor networks as a mechanism to encode heading direction information, and evidence of ring attractor-like dynamics was found in the rodent heading direction system^12,33^. However, a mechanistic understanding grounded on the neuronal connectivity that underlies such dynamics remains lacking in vertebrates.

This link between function and structure exists in the insect central complex, where elegant studies described networks that encode heading direction and constitute a neuronal implementation of a ring attractor network^4–7,9^. The level of detail being uncovered in these circuits allows for a mechanistic understanding of how a brain can integrate external and internal sensory cues, efference copies and carry out coordinate transformations that are important for behavior. The network we observe has intriguing similarities with this system, and a detailed comparative analysis of both may reveal important theoretical insights into persistent neuronal representations in general and head direction systems and ring attractors in particular^33^.

The network we describe here does not seem to be affected by visual reafference, in contrast with the insect heading direction system^4^. It is unclear whether this is due to the statistics of the visual scenery we used, as was reported in flies^34,35^, or whether it indicates that visual inputs are combined with the heading representation at a downstream level of the network. We note that the sinusoidal shape of the bump could make it ideally suited for the vector arithmetic that is required for the integration of heading direction and optic flow, as was recently shown in flies^9^, although this will need to be further investigated in future studies.

We still need to address how external cues influence this network activity to determine whether the ‘north’ of the representation points in a behaviorally relevant direction in space. The heading direction network we describe here is not modulated by visual inputs, yet such input can affect heading representation downstream of the r1π neurons. Previous work showed that zebrafish larvae might use an internal representation of heading direction to efficiently reorient in a phototaxis task^36^, and the IPN can be implicated in zebrafish directional behavior^14,15^. Information from external cues^37^ and strong excitatory drive could be provided by the dense projections from excitatory habenular neurons^38,39^, which could form synapses with an all-to-all connectivity with the dendrites of the heading direction neurons^40^. Although previously overlooked, the aHB-IPN circuit could provide an inroad to understanding the mechanisms underpinning cognitive maps in vertebrates.

## Methods

### Zebrafish husbandry

All procedures related to animal handling were conducted in accordance with protocols approved by the Technische Universität München and the Regierung von Oberbayern (animal protocol number 55-2-1-54-2532-101-12). Adult zebrafish (*Danio rerio*) of the Tupfel long fin strain were kept at 27.5–28 °C on a 14:10 hour light:dark cycle in a fish facility that provided full recirculation of water with carbon, bio- and UV filtration and a daily exchange of 12% of water. Water pH was kept at 7.0–7.5 with a 20 g l^−1^ buffer and was conductivity maintained at 750–800 µS using 100 g l^−1^. Fish were hosted in 3.5-l tanks in groups of 10–17 animals. Adults were fed Gemma Micro 300 (Skretting) and live food (*Artemia salina*) twice per day, and larvae were fed Sera Micron Nature (Sera) and ST-1 (Aquaschwarz) three times per day.

All experiments were conducted on 6–9-dpf larvae of yet-undetermined sex. The week before the experiment, one male and one female or three male and three female animals were left breeding overnight in a Tecniplast Sloping Breeding Tank or Breeding Tank. On the next day, eggs were collected in the morning, rinsed with water from the facility water system and then kept in groups of 20–40 in 90-cm petri dishes filled with 0.3× Danieau solution (17.4 mM NaCl, 0.21 mM KCl, 0.12 mM MgSO_4_, 0.18 mM Ca(NO_3_)_2_, 1.5 mM HEPES; reagents from Sigma-Aldrich) until hatching and in water from the fish facility thereafter. Larvae were kept in an incubator that maintained temperature at 28.5 °C and a 14:10 hour light:dark cycle; the solution was changed daily. At 4 or 5 dpf, animals were lightly anesthetized with tricaine mesylate (Sigma-Aldrich) and screened for fluorescence under an epifluorescence microscope. Animals positive for GCaMP6s, Dendra or mCherry fluorescence were selected for the imaging experiments.

### Transgenic animals

*Tg(gad1b/GAD67:Gal4-VP16)mpn155* (referred to as *Tg(gad1b:Gal4)*) was used for all experiments, which drives expression in a subpopulation of GABAergic cells under *gad1b* regulatory elements^41^. The animals for functional imaging and anatomical experiments were double transgenic with *Tg(UAS:GCaMP6s)mpn101* (ref. ^42^) and *Tg(UAS:Dendra-kras)s1998t* (ref. ^43^), respectively. In some anatomical experiments, the animals also had *Tg(elavl3:H2B-mCherry)*, which was generated by Tol*2* transposon-mediated transgenesis. All of the transgenic animals were also *mitfa*^*−/−*^ and thus lacked melanophores^44^.

### Lightsheet experiments

#### Preparation

For lightsheet experiments, animals were embedded in 2.2% low-melting-point agarose (Thermo Fisher) in a custom lightsheet chamber. The chamber consisted of a three-dimensional printed frame (https://github.com/portugueslab/hardware/blob/master/chambers/lightsheet_chamber_v3.stl) with a glass coverslip sealed on the side, in the position where the lateral beam of the lightsheet enters the chamber, and a square of transparent acrylic on the bottom, for behavioral tracking (Lightsheet microscope). The chamber was filled with water from the fish facility system and agarose was removed along the optic path of the lateral laser beam (to prevent scattering) and around the tail of the animal (to enable movements of the tail). In some larvae, the eyes were also freed from the agarose. After embedding, fish were left recovering for 1–6 hours before the imaging session. Before starting the imaging, we lightly tapped on the side of the chamber to select the most active fish for the experiment.

#### Lightsheet microscope

Imaging experiments were performed using a custom-built lightsheet microscope. A 473-nm wavelength laser source (modulated laser diodes; Cobolt) was used to produce an approximately 1.5-mm laser beam that was conveyed on the excitation scanning arm. The arm consisted of a pair of galvanometric mirrors that scanned vertically and horizontally, a line diffuser (Edmund Optics) to minimize stripe artifacts^45^ and a 2× telescope with a 75- and 150-mm focal distance lens (Thorlabs). The telescope expanded the beam before it entered a low numerical aperture air objective (Olympus) that then focused the beam through the lateral glass coverslip of the lightsheet chamber onto the fish. The excitation lightsheet was generated by scanning the beam on the horizontal plane at 800 Hz. A paper screen was positioned in the conjugate focal plane within the telescope lens pair to protect the eyes of the fish from the lateral scanning of the laser beam. The emitted fluorescence was collected with a 20× water immersion objective (Olympus), filtered with a 525:50 band-pass filter (AHF Analysentechnik) and focused on a CMOS camera (Hamamatsu Photonics) with a tube lens (Thorlabs).

Imaging acquisition was run using custom Python-based software^46^ to coordinate laser scanning, camera triggering and the piezo movement. The objective was moved using a sawtooth profile at a frequency of 5 Hz in most experiments (frequency was adjusted to 3 Hz in experiments where a larger vertical span was scanned). Five-volt pulses locked with the scanning profile of the piezo were sent to the camera to trigger the acquisition of each plane at a fixed vertical position during the scanning. No pulse was sent during the descending phase of scanning, when the objective would cover a large vertical span in a short time. In most experiments, eight planes were acquired over a range of approximately 80–100 µm, with slight adjustments for each fish. The resulting imaging data had a resolution of approximately 10 × 0.6 × 0.6 µm per voxel and a temporal resolution of 3–10 Hz.

#### Tail and eye tracking and stimulus presentation

To monitor tail movements during the imaging session, an infrared light-emitting diode (RS Components) was used to illuminate the larvae from above. A camera (Ximea) with a macro-objective (Navitar) was aimed at the animal through the transparent bottom of the lightsheet chamber, using a mirror placed at 45° below the imaging stage. A long-pass filter (Thorlabs) was placed in front of the camera. A projector (Optoma) was used to display visual stimuli; light from the projector was conveyed to the stage through a cold mirror that reflected the projected image on the 45° mirror placed below the stage. The stimuli were projected on a white paper screen positioned below the fish, with a triangular hole that kept the fish visible from the camera. The behavior-tracking part of the rig was very similar to the setup for restrained fish tracking described in Štih et al.^47^.

Frames from the behavioral camera were acquired at 400 Hz and tail movements were tracked online using Stytra^47^, using Stytra’s default algorithm to fit nine linear segments of the tail. During the experiment, the ‘tail angle’ quantity used for controlling the closed loop was computed (online using Stytra) as the difference between the average angle of the first two and last two segments of the tail; this was saved with the rest of the log from Stytra. For eye tracking, a video of the entire acquisition was saved to be analyzed offline (see below).

The stimulus presentation and behavior tracking were synchronized with imaging acquisition using a ZeroMQ-based trigger signal supported natively by Stytra.

### Two-photon experiments

For two-photon experiments, animals were embedded in 2% low-melting-point agarose (Thermo Fisher) in 30-mm petri dishes. The agarose around the tail, caudal to the pectoral fins, was cut away with a fine scalpel to allow for tail movement. The dish was placed onto an acrylic support with a light-diffusing screen and imaged on a custom-built two-photon microscope previously described^14^. The custom Python package brunoise was used to control the microscope hardware^48^.

Full frames were acquired every 334.51 ms in four, 0.83-μm-spaced interlaced scans, which resulted in *x*- and *y*-pixel dimensions of 0.3–0.6 μm (varying resolutions depended on field of view covered). After acquisition from one plane was complete, the objective was moved downward by 0.5–4 μm and the process was repeated.

#### Two-photon functional experiments

Visual stimuli were generated using a custom-written Python script with the Stytra package. Stimuli were projected at 60 frames per second using an Asus P2E microprojector and a red long-pass filter (Kodak Wratten 25) to allow for simultaneous imaging and visual stimulation. Fish were illuminated using infrared light-emitting diodes (850-nm wavelength) and imaged from below at up to 200 frames per second using an infrared-sensitive charge-coupled device camera (Pike F032B, Allied Vision Technologies). Tail movements were tracked online using Stytra as described for the lightsheet experiments.

#### Two-photon anatomical experiments

High resolution (0.5 × 0.5 × 0.5) two-photon stacks of the aHB and IPN were acquired from fish expressing *gad1b:Gal4* and *UAS:Dendra-kras* transgenes (*n* = 7, 6–7 dpf). The stacks were registered to one another using the Computational Morphometry Toolkit (CMTK)^49^. The transformed stacks were then averaged to generate an average brain stack showing the projections of GABAergic aHB neurons to the IPN.

### Confocal experiments

For confocal experiments, larvae were embedded in 1.5% agarose and anesthetized with tricaine mesylate (Sigma-Aldrich). Whole-brain stacks of three 7-dpf fish expressing *gad1b:Gal4*, *UAS:Dendra-kras* and *elavl3:H2B-mCherry* transgenes were acquired using a 20× water immersion objective (numerical aperture of 1.0) with a voxel resolution of 1 × 0.6 × 0.6 (LSM 880, Carl Zeiss). The stacks were registered to one another using CMTK^49^. The transformed stacks were then averaged to generate an average brain stack showing the expression pattern of *gad1b:Gal4* on top of pan-neuronal *H2B-mCherry* expression.

### EM experiments

#### SBEM dataset acquisition

Details of the larval brain SBEM dataset acquisition will be published elsewhere^50^. Briefly, a 5-dpf larval *Tg(elavl3:GCaMP5G)a4598* transgenic zebrafish was fixed with extracellular space preservation and stained as described previously^51,52^. The sample was embedded in an epoxy mixture containing 2.5% Carbon Black^53^. The brain was imaged at a resolution of 14 × 14 nm and sections were cut at a thickness of 25 nm. The long (*y*) axis of each image tile was scanned by gradually moving the stage, whereas the short axis (*x*) was scanned with the electron beam. The shape of the tile pattern was determined on the basis of a 4-μm voxel size X-ray micro-computed tomography scan (SCANCO Medical AG) of the embedded sample.

### Visual stimuli and experimental groups

In our experiments, we presented different visual stimuli to the fish but the neuronal activity we describe was not modulated by the presented visual stimuli. Therefore, for Figs. 1 and 3, we combined our observations from the following experimental conditions:

- For experiments in darkness, no visual stimuli were presented, the projector was on and a static black frame was displayed.
- For open-loop experiments, a pink noise pattern was projected and moved in *x-* and θ-planes in a path that was computed from the trajectory of a freely swimming fish taken from a previous experiment in the laboratory. The stimulus moved backward according to velocity of the fish, and rotated according to changes in its direction. As a result, the fish was presented with the optic flow that it would have perceived moving over a static pink noise pattern with that trajectory.
- For closed-loop experiments, a pink noise pattern was projected below the fish. The pattern was static if the animal did not move, and it translated backward and rotated when the fish performed spontaneous movements. The stimulus moved backward according to an estimate of the velocity of the fish computed using vigor, and rotated according to changes in its direction estimated using the swim bias. Therefore, right turns, that is, clockwise rotations of the fish, would be matched with clockwise rotations of the stimulus. The gain factor that transformed a given swim bias into an angular velocity was modulated with factors 0.5, 1 and 2 to observe if the slope of the aHB network and the estimated heading would be altered by visual feedback. An additional control gain of −1, where fish would receive a visual feedback opposite to the performed movements, was also included.
- For some directional motion experiments (*n* = 2), the animal was also shown a pink noise pattern moving in eight equally spaced directions on the plane, presented one after the other, first in a clockwise sequence (starting from forward) and then in an anticlockwise sequence.

To investigate the role of visual feedback (Fig. 4b and Extended Data Fig. 6b), we alternated 5 min of the closed-loop condition and 5 min of the open-loop condition. To address the effect of changing gains (Fig. 4b and Extended Data Fig. 6c), we performed 5-min blocks of each gain condition, with two repetitions for each condition, in the following sequence of gains: 1, 0.5, 2, 1, 0.5, 2, −1 and −1. Supplementary Fig. 6 reports all experiment protocols that were used in this study, including the conditions described above. The Stytra scripts for control of the experimental stimuli are included with the rest of the code.

### Data analysis and statistics

All data analysis was performed using Python 3.7 and Python libraries for scientific computing, in particular Numpy^54^, Scipy^55^ and Scikit-learn^56^. The Python environment replicated the analysis in the paper, which can be found in the code repository. All figures were produced using Matplotlib^57^. All statistical tests used were nonparametric, either Mann–Whitney *U* tests for unpaired comparisons (mannwhitneyu from scipy.stats) or Wilcoxon signed-rank tests for paired comparisons (wilcoxon from scipy.stats). All analysis code and source data are available. A report with the statistics of all reported numerical distributions and the exact *P* values for statistical comparisons is available in the Supplementary Information.

### Lightsheet imaging data preprocessing

Imaging stacks were saved as hdf5 files and directly input into suite2p, a Python package for calcium imaging data registration and ROI extraction^58^. We did not use suite2p algorithms for spike deconvolution. Because the planes were spaced by roughly 10 µm, we ran the detection on individual planes and did not merge ROIs across planes. Parameters used for registration and source extraction in suite2p can be found in the shared analysis code. The parameter specifying the threshold over noise that was used to detect ROIs (threshold_scaling) was adjusted differently from acquisition to acquisition to compensate for the variability in brightness that we observed from fish to fish. From the raw fluorescence traces saved from suite2p (F.npy file), change in fluorescence activity relative to baseline fluoresence (∆F/F) was calculated by taking the baseline fluorescence ΔF as the average fluorescence in a rolling window of 900 s to compensate for a small amount of bleaching that was observed in some acquisitions. The signal then was smoothed using a median filter from scipy (medfilt from scipy.signal), and *Z*-scored so that all traces were centered on zero and normalized to a standard deviation of 1. The coordinate of each ROI was taken as the centroid of its voxels. To obtain a common coordinate system for all lightsheet experiments, for each experiment, we manually defined a point, over three axes, corresponding to the midline of the fish on the anterior–inferior limit of the aHB, and transformed all coordinates so that this point was set to 0.

### Behavior data preprocessing

The behavioral data were preprocessed using the bouter package to detect swims and extract their properties^59^. First, the tail trace was processed with a function that reconstructed terminal tail segments that were mistracked during online tracking. This was performed using an interpolation based on an extrapolation from the reconstructed segments angles and the tail angles at previous time points. Then, tail angle was recomputed, and vigor was calculated as the standard deviation of the tail angle trace in a rolling window of 50 ms. Swims were defined as episodes when the vigor crossed a threshold of 0.1 for all fish. For all swims, we then computed the laterality index as the average angle of the tail during the first 70 ms of the swim. This value correlates well with the angle turned by a fish when swimming freely^14,18^. To classify right, left and forward swims, we fit a trimodal gaussian distribution to the histogram of swim laterality indexes, enforcing the two side curves to be symmetric. We then used the intercept of the central and lateral gaussians to determine the threshold used for the swim classification (± 0.239 rad).

For eye tracking, the video recording of the entire experiment was processed using DeepLabCut 2.0 (refs. ^60,61^), a Python pose estimation package based on DeeperCut^62^, to detect four points evenly spaced on each eye in each frame. Eye angle was defined as the median angle of the segments that connected the rostral-most point of the eye with all of the others. Gaze direction was defined as the average of the angles obtained for the two eyes.

### Detecting r1π neurons

r1π ROIs were first observed to be the those with the highest anticorrelation with other ROIs in the dataset. Therefore, for detection of r1π ROIs, in each experiment we computed the correlation matrix of all traces and selected ROIs that had a correlation below a given threshold with at least one other ROI in the dataset. The threshold was manually adjusted for each fish to include as many ROIs that were part of the network as possible, while excluding other signals. For all fish, this threshold was between −0.75 and −0.5. To confirm the selected ROIs were convincingly part of the r1π network and that we were including enough cells from it, we performed principal component analysis over time using only traces from the selected ROIs, and we then looked at the projection of all ROIs onto the first two PCs. When a satisfactory threshold was chosen, most included neurons formed a circular pattern in PC space (Extended Data Fig. 1g,h).

Because some additional ROIs were occasionally included, we performed an additional manual selection step on the correlation matrix of the cells. We performed optimal sorting of the traces on the basis of their angle in PC space, and then plotted the correlation matrix using the same sorting. Some traces were then excluded on the basis of the amount of discontinuity they would produce in the matrix.

We note that other approaches could be used to parse out those cells, such as restricting the anatomical location in which to find them, or including cells on the basis of the proximity to a ring fit in PC space. We used only anticorrelation and exclusion from the correlation matrix to avoid circular reasoning in our reported observations. Future investigations on this system may involve procedures that use the highly constrained dynamics of r1π neurons to isolate them from the rest of the network.

With our strategy, we detected a r1π network in approximately 20–30% of the imaged animals. In the remaining fish, behavior was sometimes very rare (a few swims over the entire experiment) or not very directional (only forward swims were performed). In other fish, even if their behavior was sufficient, the anticorrelation criterion could only locate a handful of strongly anticorrelated neurons. Although those neurons were likely to be of the described network because their activity state changed with the occurrence of directional swims, the low number of ROIs made it impossible to properly characterize their population dynamics. Finally, in some fish, the rotatory dynamics was observable only in a small temporal interval of the experiment, and they were not included in the dataset.

#### Calculation of rotated PCs

We developed a method of registering PC projections from one fish to the other in a manner that was consistent with the anatomical distribution of the r1π cells. After computing PCs over time for the r1π neurons, we (1) fit a circle to the projection of all cells using a Python hyper least square algorithm^63^ and (2) rescaled and translated the PCs to make the circle centered on (0, 0) with a radius of 1.

Then, we computed a weighted average across all of the vectors representing ROIs in this two-dimensional space, weighted by their location in the rostrocaudal and the left–right anatomical axes (Supplementary Fig. 1a,b). As a result, we obtained two vectors, one pointing in the direction of the most-rostral ROIs, and the other in the direction of the rightmost ROIs. We then rotated and flipped each fish’s projection so that those two axes matched across fish, that is, the sum of the absolute magnitude of the two angles’ distances abs(*θ*_1_) + abs(*θ*_2_) visible in Supplementary Fig. 1b was minimized (Supplementary Fig. 1a,b). We call the axes of this space rotated PCs (rPCs).

After calculating rPCs for an experiment, all ROIs were assigned an angle *α* on the basis of their position over the circle in rPC space. The convention used for the angle was as follows:

- *α* ∈ (−*π,* *π*]
- Caudal neurons had *α* = 0
- *α* increased when moving clockwise in the anatomical location of the neurons

Therefore, looking from above the horizontal plane, leftmost ROIs had an *α* of π/2, and rightmost ROIs had an *α* of −π/2 (Supplementary Fig. 1c).

To test the hypothesis that the network is anatomically organized, we used the Fisher–Lee definition of a circular correlation coefficient^64^. We also fit a sinusoidal curve to the distribution of the ROI’s left–right and anterior–posterior coordinates over the ROI’s angle in rPC space, and compared the fit residuals to the residuals computed over a shuffle computed by randomly reassigning ROI coordinates (Extended Data Fig. 1i).

#### Network phase calculation

We derived the phase *φ*(*t*) to describe which part of the circle in rPC space was the most active at every time point (Supplementary Fig. 3a and Supplementary Video 2). For each frame, we computed a vector average **v** of all the *n* ROI vectors **rPC**_*i*_ in the two-dimensional rPC space, weighted by the state of activation of each ROI *f(t)* (the ∆F/F at time *t*):

$$\mathbf{v}\left( t \right) = \frac{1}{n}\mathop {\sum }\limits_{i = 1}^n f_i\left( t \right){\mathbf{rPC}}_{i}$$

Note that for this vector averaging, the ∆F/F of all ROIs at time *t* were clipped to their second and 98th percentiles and normalized to have mean 0 across the ROIs at every time point:

$$\mathop {\sum }\limits_{i = 1}^n f_i\left( t \right) = 0$$

where **rPC**_*i*_ is the two-dimensional vector of **rPC** scores for the *i*th neuron, and *f*_*i*_*(t)* its (normalized) ∆F/F at time *t*.

The network phase *φ*(*t*) is then defined as the angle *φ*(*t*) subtended by this vector **v**(*t*) subject to the same conventions as *α*_*i*_ defined above (Supplementary Fig. 1c):

- *φ* = *0* corresponds to caudal neurons being active
- Increments in *φ* correspond to activity rotating clockwise, and decrements of *φ* to activity rotating anticlockwise

Therefore, *φ* = *0* corresponded to the activation of the network in the rostral part, *φ* = π/2 to activation of the left part, *φ* = π/−π to activation in the rostral part and *φ* = −2π to activation in the right part (Supplementary Fig. 1c).

For all further analyses, the unwrapped or cumulative phase was used (numpy.unwrap function), that is, every discontinuity at π/−π was removed by adding to parts of the trace an offset 2π*k* for some integer *k*.

#### Calculation of average activity profile

To estimate the average activation profile of the network across the ring of neurons (Supplementary Fig. 3), we first interpolated the neuron’s traces to a matrix spanning the interval −π to +π in 100 bins (Supplementary Fig. 4a). We then circularly shifted each column of the matrix so that the phase, and hence the network activation peak, was always positioned at the center of the matrix (Fig. 2a). Finally, we calculated the average and standard deviation of the matrix across the time axis. To ensure the result was not the consequence of the resampling procedure, we also performed the circular shift of the raw matrix of traces, sorted according to neurons’ *α*, and we obtained consistent results (Supplementary Fig. 4b). The interpolation used for the average activation profile calculation has not been used for other visualizations of raw traces, such as Figs. 1, 3 and 5 and Extended Data Fig. 3.

#### Estimated heading calculation and correlation with phase

To compute estimated heading for the analysis reported in Fig. 3, we estimated the instantaneous angular velocity as the laterality index value for each individual swim (Behavior data preprocessing) and integrated it over time to obtain an estimated heading direction for the head-restrained fish.

We note that, although the relationship between the laterality index and the fish orientation change in freely swimming animals is highly linear, the slope of the linearity is not necessarily one. Furthermore, the precise extent of the tail that is tracked, the embedding procedure and the fact that the head is immobilized in agarose for our head-restrained imaging experiments are all parameters that can affect the precise kinematics of the tail movements and make a precise numerical comparison between head-restrained and freely swimming experiments difficult. Therefore, we did not aim at reconstructing a fully realistic estimated heading direction and we relied on quantifications that either captured only the correlation between estimated heading changes and network phase changes, or quantified the slope coefficient between the two quantities in relative comparisons within one experiment (for the visual feedback and gain change experiments).

For the results reported in Fig. 3f, we calculated, for each fish, the correlation between heading and phase in a rolling window of 300 s (ten overlaps for each window), and the same correlation but using a nonoverlapping 5-min epoch of the heading trace for the shuffle distribution. The moments reported in Fig. 3f refer to this population of intervals and shuffle intervals for each fish.

#### Swim-triggered and saccade-triggered analyses

For the directional swim-triggered and saccade-triggered analysis of Fig. 3c and Extended Data Fig. 7i, we cropped, for each fish, the phase around each event, computed a fish average for all curves with at least *n* = 3 cropped samples and we subtracted the mean of the 10-second interval before the event.

#### Heading and phase slope fitting for visual feedback experiments

In the experiments reported in Fig. 4a–c, we wanted to quantify whether the presence of closed-loop visual feedback or the effect of different gain parameters of the closed-loop visual feedback had an effect on the relationship between the change in heading and the phase of the network. Because swims often happen in sequences and the average network phase change seemed to plateau after approximately 10 s from the focal swim, we observed the relationship between the amount of phase changed in a window between 15 and 20 s after the swim (Δphase_15–20s_), and the amount of estimated heading change in the same interval (Δheading_15–20s_) (which will potentially accumulate the effect of other swims in the sequence). The choice of window size was arbitrary, and all of the results are similar using other intervals in the 5–20-s range. To quantify the heading and phase relationship, we performed linear regression on the points (Δphase_15__–__20s_, Δheading_15__–__2__0__s_) points for all swims in each experimental condition (Extended Data Fig. 6a shows this calculation for all fish) and we compared the values of the regression slope across conditions (Fig. 4a,b).

#### Left and right swim and gaze angle regression

We performed a regression analysis to understand whether there was activity in the neural region related to left and right swims. Using an exponential decay function, we established a set of regressors by convolving an array that was zero everywhere and one in correspondence with either left or right swims (Extended Data Fig. 7a) along with an array for gaze direction (Extended Data Fig. 7e). The time constant used was 3 s; although this value was higher than the GCaMP6s time constant, it was chosen because it more closely matched the experimentally observed curves. The exact value of the time constant was not critical for the reported results. Each cell’s fluorescence trace was then correlated with both regressors, and the correlation values were used for the analysis and visualizations in Fig. 4c and Extended Data Fig. 7b–d,g,h. In the maps of Fig. 4c and Extended Data Fig. 7c,d, left and right swim-related cells were classified as such if their correlation with the left or right swim regressor was more than 0.7, and correlation with the other regressor was less than 0.7.

#### Multilinear regression of eye and tail to network phase

To address the relationship between network phase and eye motion, we used gaze direction, computed as the average between the angles of the two eyes. For regression analysis, we used gaze velocity or the instantaneous fish angular velocity estimated from the swim laterality indexes (both convolved using the same τ as in left and right swim regression), either alone or in combination, to fit the temporal derivative of the (unwrapped) network phase. Because a multilinear regression will probably outperform the regression using only one of the two regressors just by overfitting, we crossvalidated the analysis by first calculating the regression values on a randomly drawn epoch of 5 min of the experiment, and calculated the correlation of the phase derivative and the predicted phase derivative in a test 5-min epoch, drawn randomly by making sure it did not overlap with the fit window. The random sampling procedure was repeated 500 times to obtain 500 draws, and the plot in Fig. 3 freports, for each fish, the moments of the correlation values obtained over the population of such draws.

#### SBEM data skeletonization

The first reconstructions of cells in the aHB with processes in the IPN were observed by seeding dendrites or axons in the IPN for reconstruction and then tracing toward the somata in the aHB, in the context of a (still unpublished) broader reconstruction effort. The IPN location in the EM stack was first inferred by the recognizable organization of the neuropil and cell somata in the rhombomere 1 ventral region. Then, the location was confirmed by tracing axons that could be reconstructed back to the habenulae through a long bundle of fibers unambiguously identifiable as the fasciculus retroflexus by its course (unpublished data). After these first observations, additional cells with somata in the aHB were seeded on the basis of the similarity of their processes with already reconstructed cells. Skeletonization was performed manually by a team of annotators at ariadne.ai AG. Annotators were instructed to flag difficult locations without extending the skeleton at those locations, and to stop tracing after a total time of 2 hours was reached. At that point, or when a cell was completed, a quality check was performed by an expert annotator. Difficult locations were then decided by the expert, and sent back to the annotator team for additional tracing if necessary. This procedure was iterated until all cells were fully traced. The skeletons were then annotated to distinguish the dendrite and the axon by their morphological features (process thickness and presence of presynaptic boutons) independently by Ariadne expert annotators or the authors, with convergent results. All further analyses and quantifications of the reconstructions were performed using Python. To calculate the centroid position of dendrite and axon for the analyses in Fig. 6a,b, we took the average coordinate of the coordinates (in IPN reference space) of all of the branching points of dendrites and axons. To generate the distance plot in the bottom of Fig. 6d, we calculated, for every branching point of every neuron, the distance along the frontal and sagittal axis of all of the other branching points (of both axons and dendrites) and showed the distribution of such distances.

Although the SBEM dataset comes from a younger animal, the neuronal morphologies we observed appear to be mature, with extensive and structured arborizations; the fiber tracts that connect the r1π neurons to the IPN, very prominent in the EM reconstructions, also appear very clearly in confocal stacks from the 7–9-dpf animal. Moreover, parts of the IPN circuitry, such as the axonal arborization from the habenulae, appear to be morphologically and functionally mature even at 5 dpf (refs. ^38,40^). Therefore, it is reasonable to assume that the morphologies observed in the 5-dpf animal from the SBEM dataset would be maintained at the later stages of functional experiments.

#### Anatomical registrations

To work with the anatomical spaces and their annotations, we used the BrainGlobe bg-atlasapi package^65^ and either the larval zebrafish brain reference mapZebrain^66^, or a custom laboratory reference of the aHB and IPN region created by morphing together stacks from different lines using either dipy^67^ or CMTK^49^. To visualize functional data in the references, an average anatomy was computed after centering all stacks with the centering point described in Lightsheet imaging data preprocessing, and then a manual affine registration was performed to the IPN reference. A similar procedure was used to map the EM data. From the skeletons, a density stack was computed in which the shape and features of the IPN were prominently visible. An affine matrix transformation was found to match this stack on the IPN reference, and this was used for transforming the neuron’s coordinates. The masks delimiting the IPN and the dIPN were drawn in the IPN reference atlas by considering the localization of habenular axon afferents to the region.

#### Two-dimensional autocorrelation of neuronal activity

For the plots reported in Fig. 6, two-photon microscopy images from a single plane in the IPN were aligned to the frontal and sagittal axes of the brain. The dIPNs in the images were masked by manual drawing. The area inside of the mask was divided into 3.5 × 3.5-μm^2^ bins. The average fluorescence signal at each bin was *Z*-scored. For each bin, the Pearson correlation of the signal traces between the focal bin and all other bins was computed and sorted in two dimensions by the distances between two bins in the frontal and sagittal axes. The correlation coefficients at the same distance were averaged across bins for each animal, and then averaged across all animals.

### Reporting summary

Further information on research design is available in the Nature Portfolio Reporting Summary linked to this article.

## Online content

Any methods, additional references, Nature Portfolio reporting summaries, source data, extended data, supplementary information, acknowledgements, peer review information; details of author contributions and competing interests; and statements of data and code availability are available at 10.1038/s41593-023-01308-5.

## Supplementary information

Supplementary Information Supplementary Figs. 1–7, legends for Videos 1–3 and a statistical summary log.

Reporting Summary

Supplementary Video 1 Raw activity of all neurons in the example fish reported in Fig. 1. The pink contours highlight the r1π neurons in the aHB.

Supplementary Video 2 Animation showing phase calculation as in Supplementary Fig. 3a.

Supplementary Video 3 Rendering of aHB neuron morphologies mirrored bilaterally, color coded by a fictive angle computed on the basis of their dendrite position.

## Data Availability

All of the source data used in the functional imaging analysis (raw ∆F/F traces, ROI maps/coordinates, behavioral traces and stimulus log from Stytra) and for the anatomical observations (confocal/two-photon stacks, EM skeletons) are available at 10.5281/zenodo.6847130. Raw functional imaging data can be made available upon request.

## Code availability

All of the scripts for stimuli generation, data preprocessing, analysis and figure generation are available at 10.5281/zenodo.7715409.

## References

[1] Moser EI, Kropff E, Moser MB (2008). Place cells, grid cells, and the brain’s spatial representation system. Annu. Rev. Neurosci..

[2] Taube JS, Muller RU, Ranck JB (1990). Head-direction cells recorded from the postsubiculum in freely moving rats. I. Description and quantitative analysis. J. Neurosci..

[3] Taube JS (2007). The head direction signal: origins and sensory-motor integration. Annu. Rev. Neurosci..

[4] Seelig JD, Jayaraman V (2015). Neural dynamics for landmark orientation and angular path integration. Nature.

[5] Green J (2017). A neural circuit architecture for angular integration in Drosophila. Nature.

[6] Kim SS, Rouault H, Druckmann S, Jayaraman V (2017). Ring attractor dynamics in the Drosophila central brain. Science.

[7] Fisher YE (2019). Sensorimotor experience remaps visual input to a heading-direction network. Nature.

[8] Suver MP (2019). Encoding of wind direction by central neurons in Drosophila. Neuron.

[9] Lyu C, Abbott LF, Maimon G (2022). Building an allocentric travelling direction signal via vector computation. Nature.

[10] Sharp PE, Blair HT, Cho J (2001). The anatomical and computational basis of the rat head-direction cell signal. Trends Neurosci..

[11] Clark BJ, Taube JS (2009). Deficits in landmark navigation and path integration after lesions of the interpeduncular nucleus. Behav. Neurosci..

[12] Clark BJ, Sarma A, Taube JS (2009). Head direction cell instability in the anterior dorsal thalamus after lesions of the interpeduncular nucleus. J. Neurosci..

[13] Quina LA, Harris J, Zeng H, Turner EE (2017). Specific connections of the interpeduncular subnuclei reveal distinct components of the habenulopeduncular pathway. J. Comp. Neurol..

[14] Dragomir EI, Štih V, Portugues R (2020). Evidence accumulation during a perceptual decision task revealed by whole-brain imaging. Nat. Neurosci..

[15] Cherng BW, Islam T, Torigoe M, Tsuboi T, Okamoto H (2020). The dorsal lateral habenula-interpeduncular nucleus pathway is essential for left-right-dependent decision making in Zebrafish. Cell Rep..

[16] Skaggs WE, Knierim JJ, Kudrimoti HS, McNaughton BL (1995). A model of the neural basis of the rat’s sense of direction. Adv. Neural Inf. Process. Syst..

[17] Zhang K (1996). Representation of spatial orientation by the intrinsic dynamics of the head-direction cell ensemble: a theory. J. Neurosci..

[18] Huang K-HH, Ahrens MB, Dunn TW, Engert F (2013). Spinal projection neurons control turning behaviors in zebrafish. Curr. Biol..

[19] Yoder RM, Taube JS (2014). The vestibular contribution to the head direction signal and navigation. Front. Integr. Neurosci..

[20] Wolf S (2017). Sensorimotor computation underlying phototaxis in zebrafish. Nat. Commun..

[21] Ramirez AD, Aksay ERF (2021). Ramp-to-threshold dynamics in a hindbrain population controls the timing of spontaneous saccades. Nat. Commun..

[22] Dunn TW (2016). Neural circuits underlying visually evoked escapes in larval zebrafish. Neuron.

[23] Chen X (2018). Brain-wide organization of neuronal activity and convergent sensorimotor transformations in larval zebrafish. Neuron.

[24] Allen GV, Hopkins DA (1989). Mamillary body in the rat: yopography and synaptology of projections from the subicular complex, prefrontal cortex, and midbrain tegmentum. J. Comp. Neurol..

[25] Wirtshafter D, Stratford TR (1993). Evidence for GABAergic projections from the tegmental nuclei of Gudden to the mammillary body in the rat. Brain Res..

[26] Contestabile RA, Flumerfelt BA (1981). Afferent connections of the interpeduncular nucleus and the topographic organization of the habenulo‐interpeduncular pathway: an HRP study in the rat. J. Comp. Neurol..

[27] Groenewegen HJ, Ahlenius S, Haber SN, Kowall NW, Nauta WJH (1986). Cytoarchitecture, fiber connections, and some histochemical aspects of the interpeduncular nucleus in the rat. J. Comp. Neurol..

[28] Liu R, Chang L, Wickern G (1984). The dorsal tegmental nucleus: an axoplasmic transport study. Brain Res..

[29] Herrick, C. J. *The Brain of the Tiger Salamander, Ambystoma tigrinum* (Univ. of Chicago Press, 1948).

[30] Iwahori N, Nakamura K, Kameda S, Tahara H (1993). Terminal patterns of the tegmental afferents in the interpeduncular nucleus: a Golgi study in the mouse. Anat. Embryol. (Berl.)..

[31] Hansel, D. & Sompolinsky, H. in *Methods in Neuronal Modeling: From Synapse to Networks* 2nd edn (eds Koch, C. & Segev, I.) Ch. 13 (MIT Press, 1998).

[32] Hulse BK, Jayaraman V (2020). Mechanisms underlying the neural computation of head direction. Annu. Rev. Neurosci..

[33] Chaudhuri R, Gerçek B, Pandey B, Peyrache A, Fiete I (2019). The intrinsic attractor manifold and population dynamics of a canonical cognitive circuit across waking and sleep. Nat. Neurosci..

[34] Kim SS, Hermundstad AM, Romani S, Abbott LF, Jayaraman V (2019). Generation of stable heading representations in diverse visual scenes. Nature.

[35] Haberkern, H. et al. Maintaining a stable head direction representation in naturalistic visual environments. Preprint at *bioRxiv*10.1101/2022.05.17.492284 (2022).

[36] Chen X, Engert F (2014). Navigational strategies underlying phototaxis in larval zebrafish. Front. Syst. Neurosci..

[37] Dreosti E, Vendrell Llopis N, Carl M, Yaksi E, Wilson SW (2014). Left-right asymmetry is required for the habenulae to respond to both visual and olfactory stimuli. Curr. Biol..

[38] Hong E (2013). Cholinergic left-right asymmetry in the habenulo-interpeduncular pathway. Proc. Natl Acad. Sci. U S A.

[39] Villani L, Battistini S, Bissoli R, Contestabile A (1987). Cholinergic projections in the telencephalo-habenulo-interpeduncular system of the goldfish. Neurosci. Lett..

[40] Bianco IH, Wilson SW (2009). The habenular nuclei: a conserved asymmetric relay station in the vertebrate brain. Philos. Trans. R. Soc. Lond. B. Biol. Sci..

[41] Förster D (2017). Genetic targeting and anatomical registration of neuronal populations in the zebrafish brain with a new set of BAC transgenic tools. Sci. Rep..

[42] Thiele TR, Donovan JC, Baier H (2014). Descending control of swim posture by a midbrain nucleus in zebrafish. Neuron.

[43] Arrenberg AB, Del Bene F, Baier H (2009). Optical control of zebrafish behavior with halorhodopsin. Proc. Natl Acad. Sci. U S A.

[44] Lister JA (1999). nacre encodes a zebrafish microphthalmia-related protein that regulates neural-crest-derived pigment cell fate. Development.

[45] Taylor MA, Vanwalleghem GC, Favre-Bulle IA, Scott EK (2018). Diffuse light-sheet microscopy for stripe-free calcium imaging of neural populations. J. Biophotonics.

[46] Štih, V., Asua, D., Petrucco, L., Puppo, F. & Portugues, R. Sashimi (v0.2.1). *Zenodo*10.5281/zenodo.5932227 (2020).

[47] Štih V, Petrucco L, Kist AM, Portugues R (2019). Stytra: an open-source, integrated system for stimulation, tracking and closed-loop behavioral experiments. PLoS Comput. Biol..

[48] Štih, V., Paoli, E., Wu, Y.K., van Beelen, N., Asua, D. & Portugues, R. portugueslab/brunoise: Alpha (0.1). *Zenodo*10.5281/zenodo.4122063 (2020).

[49] Rohlfing T, Maurer CR (2003). Nonrigid image registration in shared-memory multiprocessor environments with application to brains, breasts, and bees. IEEE Trans. Inf. Technol. Biomed..

[50] Svara F (2022). Automated synapse-level reconstruction of neural circuits in the larval zebrafish brain. Nat. Methods.

[51] Svara FN, Kornfeld J, Denk W, Bollmann JH (2018). Volume EM reconstruction of spinal cord reveals wiring specificity in speed-related motor circuits. Cell Rep..

[52] Briggman KL, Helmstaedter M, Denk W (2011). Wiring specificity in the direction-selectivity circuit of the retina. Nature.

[53] Nguyen HB (2016). Conductive resins improve charging and resolution of acquired images in electron microscopic volume imaging. Sci. Rep..

[54] Harris CR (2020). Array programming with NumPy. Nature.

[55] Virtanen P (2020). SciPy 1.0: fundamental algorithms for scientific computing in Python. Nat. Methods.

[56] Pedregosa F (2011). scikit-learn: machine learning in Python. J. Mach. Learn. Res..

[57] Hunter JD (2007). Matplotlib: a 2D graphics environment. Comput. Sci. Eng..

[58] Pachitariu, M. et al. Suite2p: beyond 10,000 neurons with standard two-photon microscopy. Preprint at *bioRxiv*10.1101/061507 (2016).

[59] Štih, V., Petrucco, L., Prat, O., Lavian, H. & Portugues, R. Bouter (v20.2.0). *Zenodo*10.5281/zenodo.5931684 (2020).

[60] Mathis A (2018). DeepLabCut: markerless pose estimation of user-defined body parts with deep learning. Nat. Neurosci..

[61] Nath T (2019). Using DeepLabCut for 3D markerless pose estimation across species and behaviors. Nat. Protoc..

[62] Insafutdinov, E., Pishchulin, L., Andres, B., Andriluka, M. & Schiele, B. DeeperCut: a deeper, stronger and faster multi-person pose estimation model. In *Computer Vision – ECCV 2016. Lecture Notes in Computer Science* Vol. 9906 (eds Liebe, B. et al.) 34–50 (Springer, 2016).

[63] Kanatani K, Rangarajan P (2011). Hyper least squares fitting of circles and ellipses. Comput. Stat. Data Anal..

[64] Fisher NI, Lee AJ (1983). A correlation coefficient for circular data. Biometrika.

[65] Claudi F (2020). BrainGlobe Atlas API: a common interface for neuroanatomical atlases. J. Open Source Softw..

[66] Kunst M (2019). A cellular-resolution atlas of the larval zebrafish brain. Neuron.

[67] Garyfallidis E (2014). Dipy, a library for the analysis of diffusion MRI data. Front. Neuroinform..

