# Supplementary material for: Neural dynamics and architecture of the heading direction circuit in zebrafish
Source: Nat Neurosci. 2023 Apr 24;26(5):765–73. doi: 10.1038/s41593-023-01308-5 (PMC10166860; doi:10.1038/s41593-023-01308-5)
Supplement: Supplementary file 1 — Supplementary Figs. 1–7, legends for Videos 1–3 and a statistical summary log. [file 41593_2023_1308_MOESM1_ESM.pdf]

nature neuroscience

Article

https://doi.org/10.1038/s41593-023-01308-5

Neural dynamics and architecture of the heading direction circuit in zebrafish

In the format provided by the authors and unedited

Supplementary information

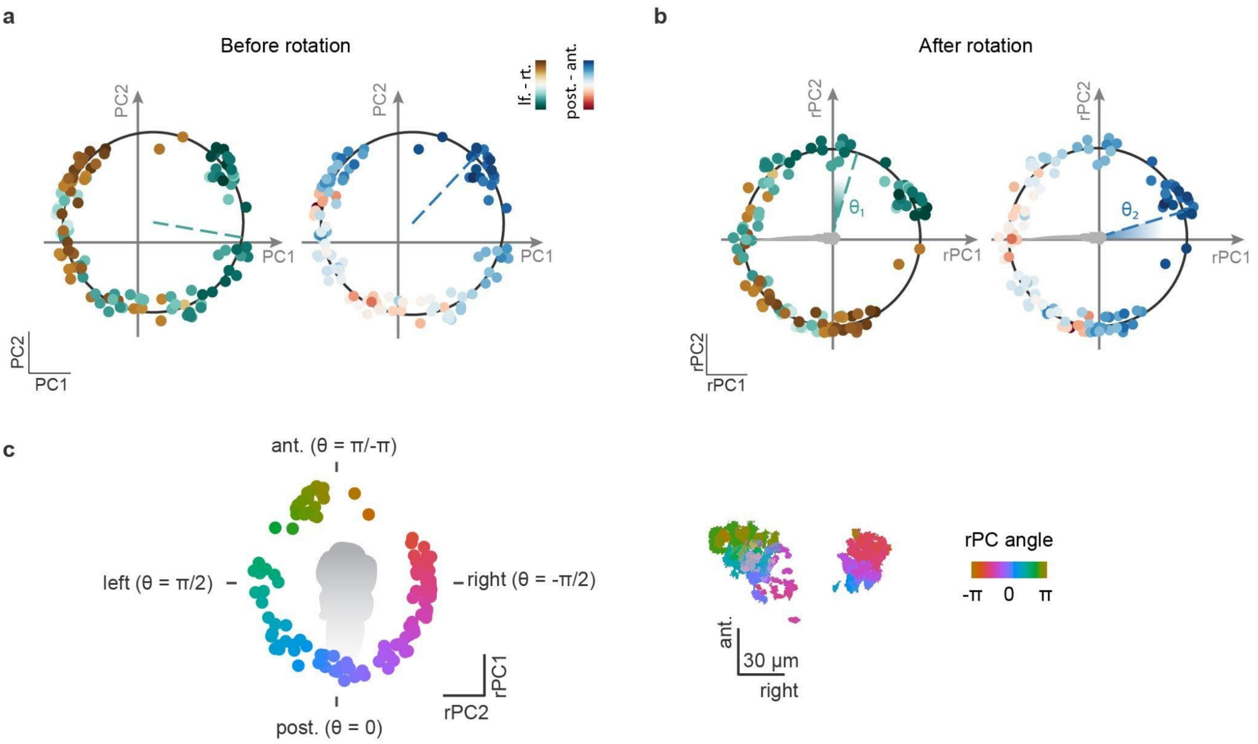

Supplementary figure 1: Anatomical organization of r1π neurons angles. **a**, Co-registration of PC projections. Vector averages of the individual neurons vectors weighted by the anatomical location of ROIs on the frontal axis (left) and sagittal axis (right) were used to map the anatomical axes on the PC space (dashed lines). **b**, Both vectors were then averaged and used to find a rotation (and in some case a flip) that would minimize the sum of the two angles θ_1 and θ_2. **c**, Illustration of the convention used for defining angles in the paper. Left: ROIs in rPC space, with labeled angles and corresponding anatomical position of the ROIs. Right: the anatomy of the same ROIs. Both plots are color-coded by angle in rPC space.

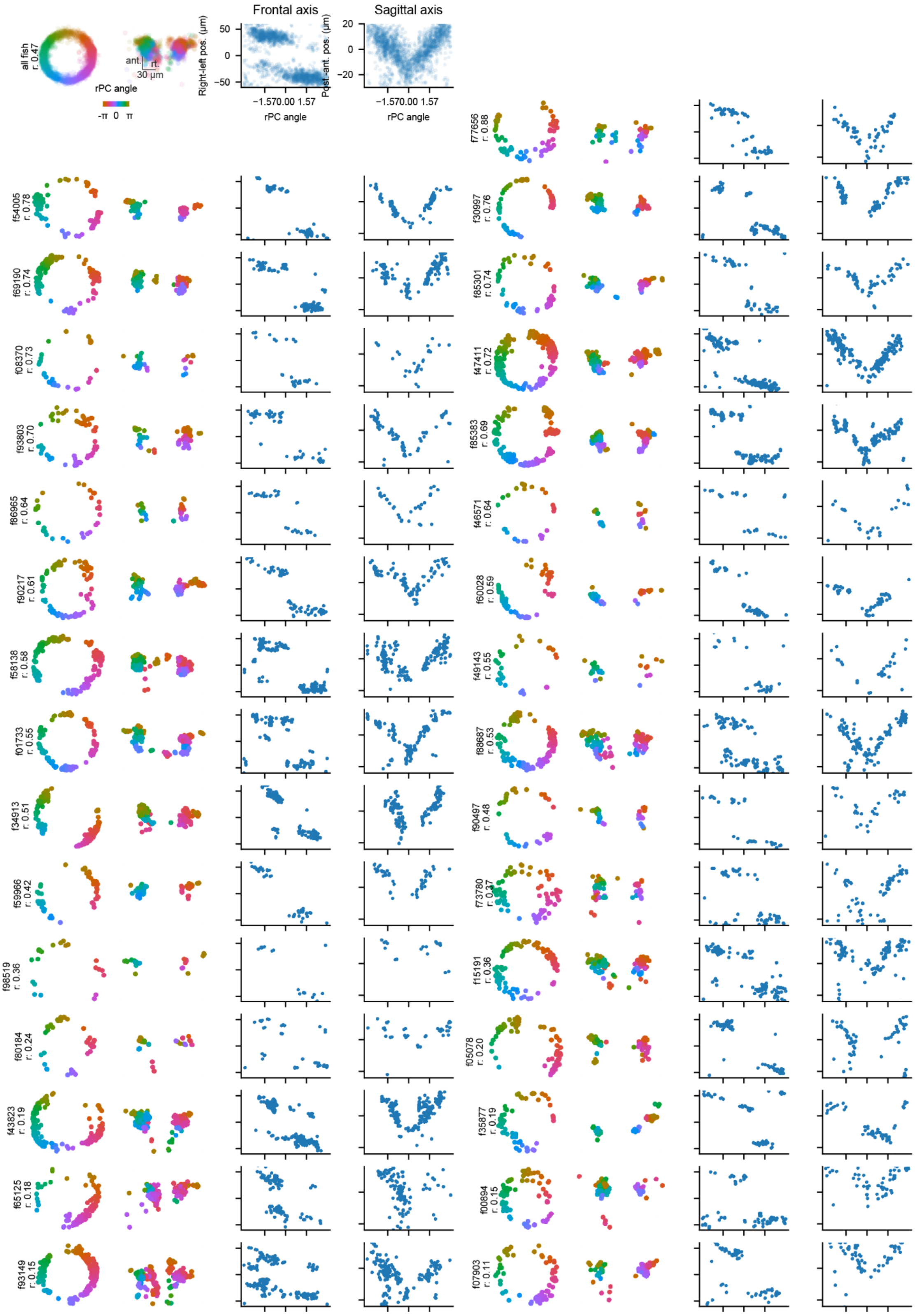

Supplementary figure 2: Anatomical organization of r1π neurons angles. Figures for individual fish. Same plots reported in Figure 1g and Extended Data Figure 1i-j, split for individual fish.

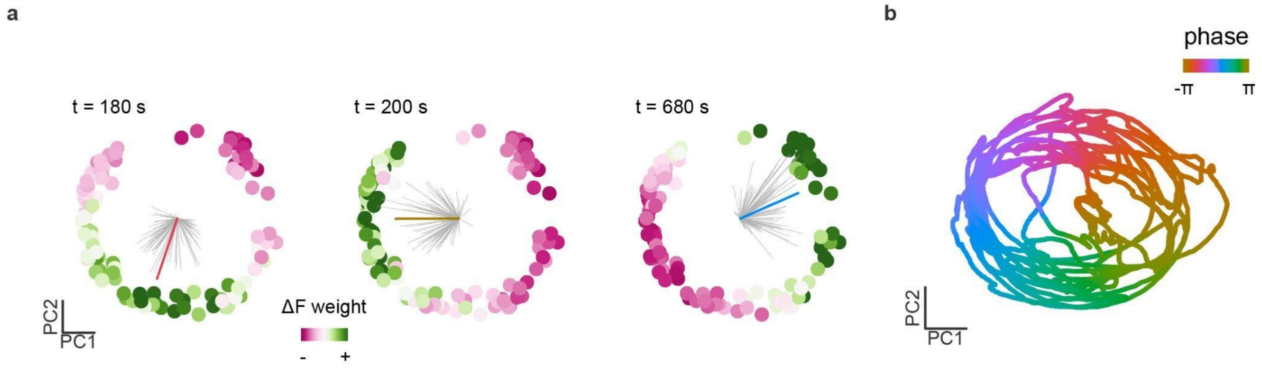

Supplementary figure 3: Network phase calculation. **a**, Network phase was computed as the angle of the vector average over all neuron projections in rPC space weighted by their (normalized) activation. In the plot, left, center, and right panels show the rPC projections color-coded by the state of activation of neurons at three different time points. Gray lines show the weighted vector of each neuron, and the thick line their average, color-coded by their angle. **b**, Trajectory of the network in PC-reduced phase space, color-coded by the network phase.

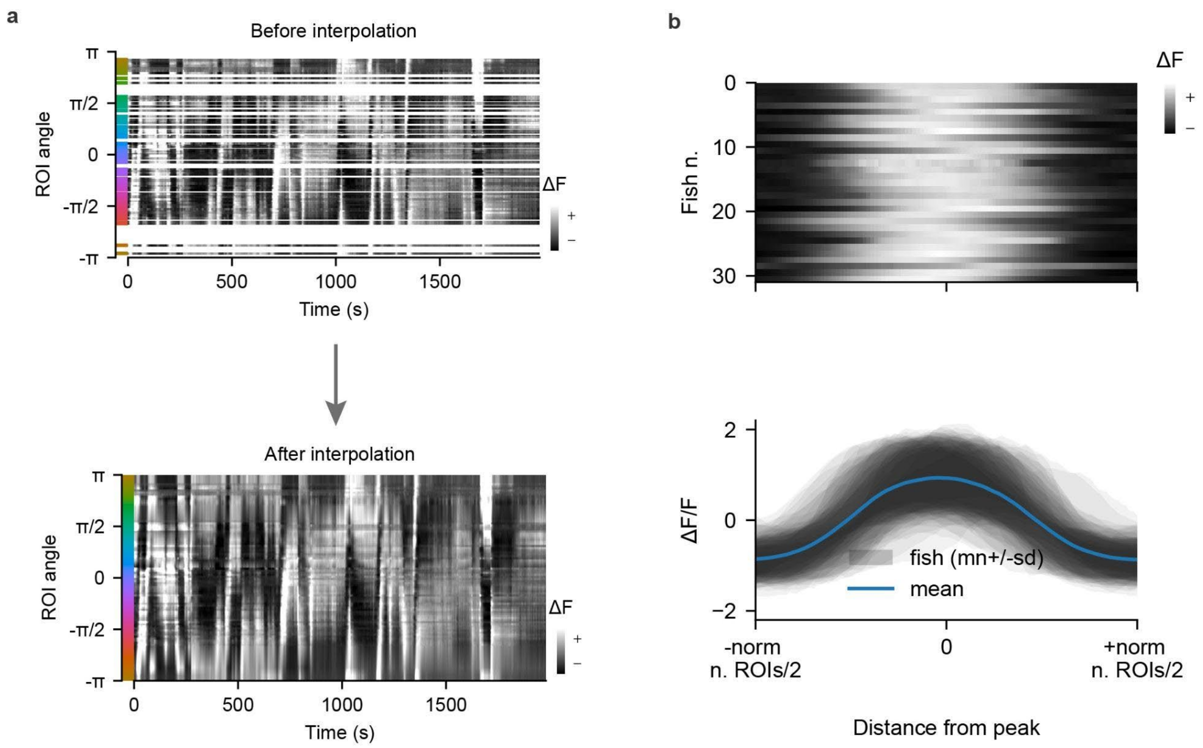

Supplementary figure 4: Network activity profile. **a**, Interpolation of network activity from neuron angles. (top) Traces of individual neurons sorted and spaced in abscissae using their angle θ. The colors on the left map neuron angles. (bottom) The same activity, after interpolating the activation between -π and π. **b**, Average activation profile for all fish in the dataset (n = 31 fish). (top) Matrix showing the average activation profile for all fish in the dataset and (bottom) mean ± std over time for each fish (shaded areas) and population average (blue). Same plots of Figure 2, but computed by phase-zeroing in the traces matrix without interpolation. The average shape of the bump is very consistent.

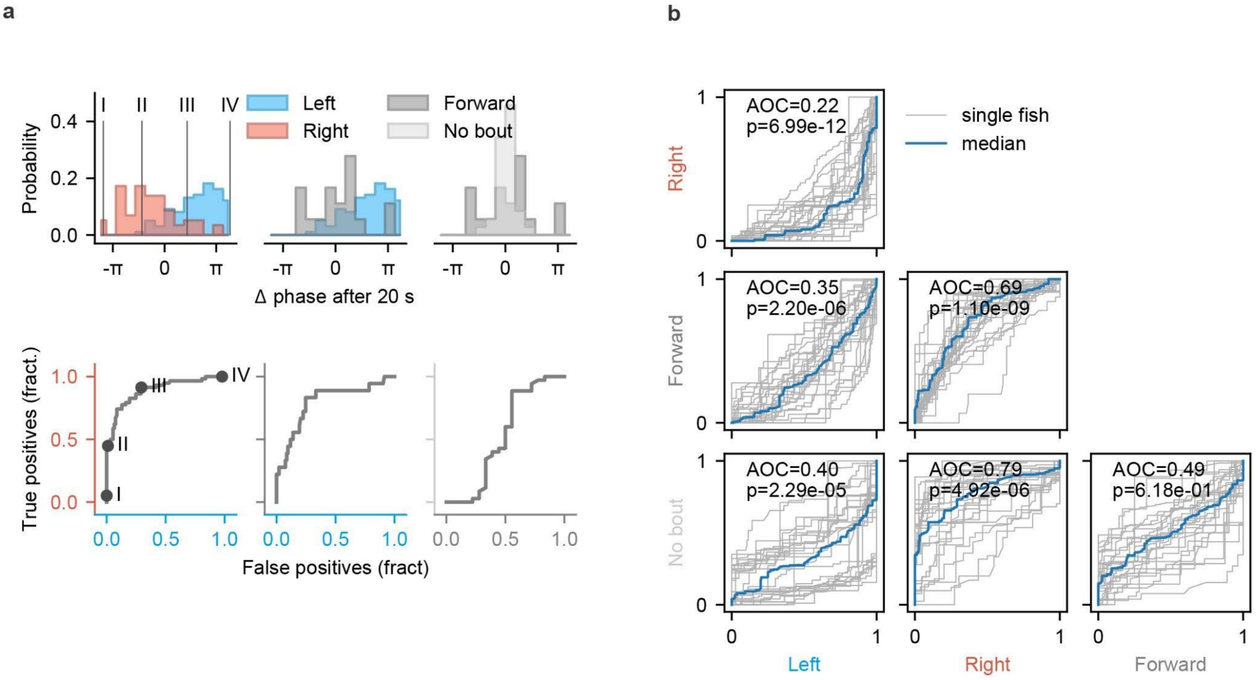

Supplementary figure 5: Stability of bump and phase dynamics. **a**, example of ROC curve calculation from the histograms of phase change after 20 s from a swim, given the directionality of the swim, (above). Below, ROC curve calculated from the distributions above, with four choices of thresholds matched in the first plots above and below. **b**, distributions of ROC curves between each condition pair for every fish (gray lines) and their averages (blue line). P-values are calculated with a Wilcoxon Rank Sum test against the null hypothesis that they are all 0.5.

a

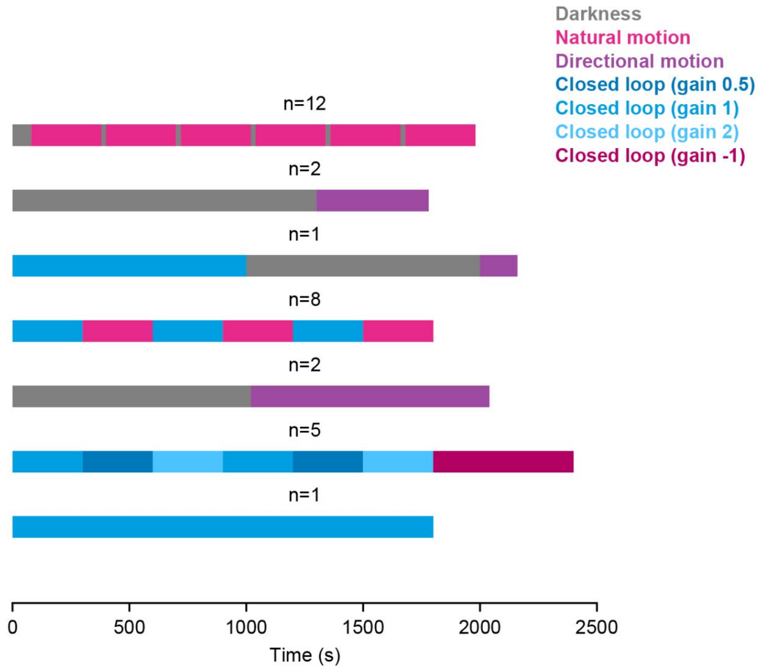

Supplementary figure 6: Schema of experimental protocols. **a**, Color-coded description of the sequences of blocks used in the various experiments described in the paper.

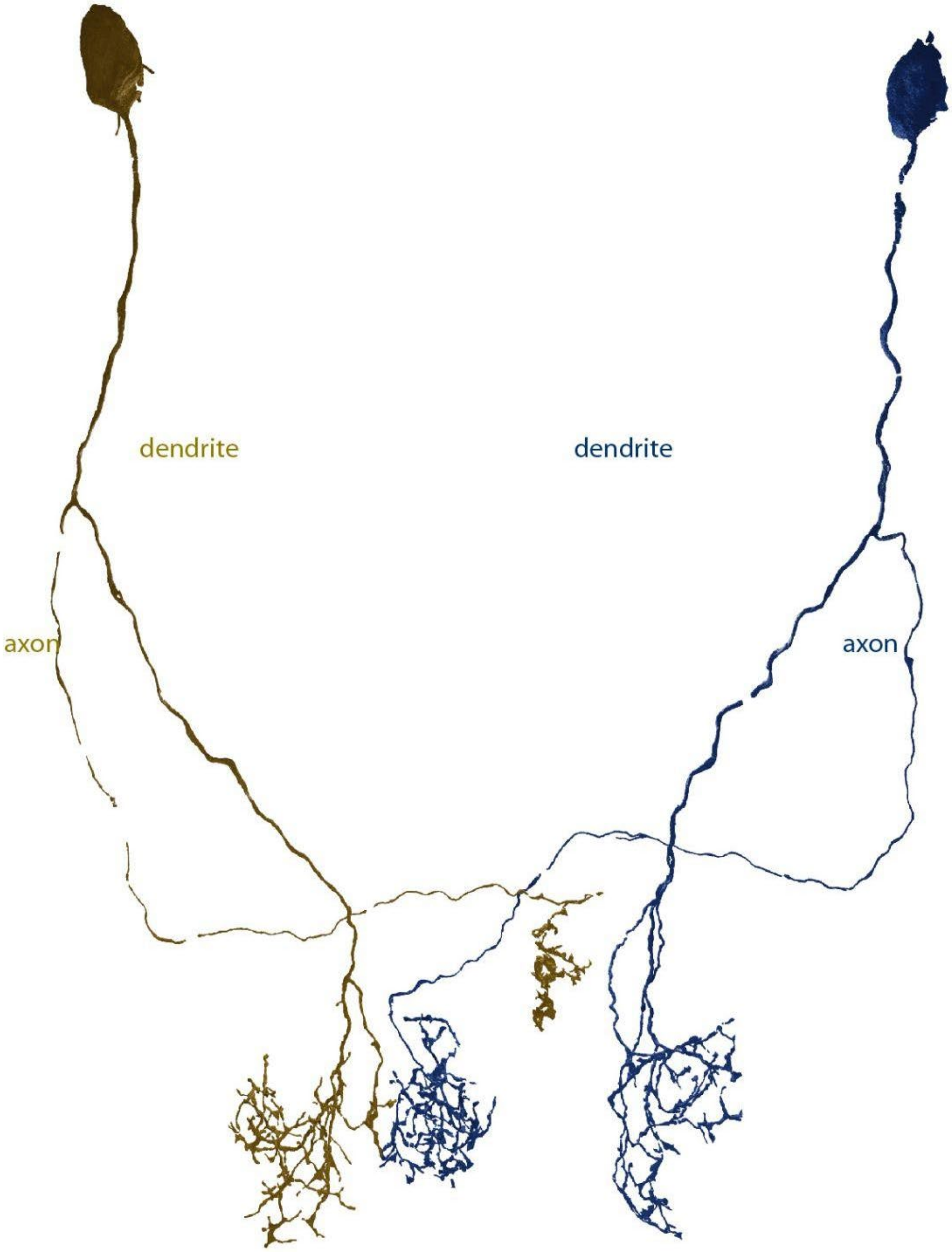

Supplementary figure 7: r1π neurons extend an ipsilateral dendritic shaft and a contralateral axonal shaft. Full segmentation of two example neurons from the EM dataset, showing their morphological features. The ipsilateral shaft (the dendrite) is thicker than the contralateral one (the axon).

Supplementary Video 1: Raw activity of all neurons in the example fish reported in Fig 1. The pink contours highlight the r1π neurons in the aHB.

Supplementary Video 2: Animation showing phase calculation as in Fig S3a.

Supplementary Video 3: rendering of aHB neuron morphologies mirrored bilaterally, color-coded by a fictive angle computed based on their dendrite position.

Statistical summary log

Those are the values generated by running the deposited analysis code with the deposited data for all the distributions and the statistical comparisons in the paper. For a thorough description of each item on the log please refer to the deposited data folder. The following summary was generated with version 1.0.1 of the code.

```
[log_info]
created = 2023-03-10 02:22:44
dataset = {'210728_f1eyes_clol', '210810_f0_cwccw', '210508_f1_spont', '210601_f0_natmov_noeyes', '210716_f7_clol', '210805_f3_cwccw', '210924_f2_gainmod', '210602_f0_natmov_spont', '210511_f1_natmov', '211207_f3eyes_natmov', '210601_f3_natmov_spont', '210924_f1_gainmod', '210715_f5_clol', '210511_f0b_natmov', '210601_f0_2dvr_eyes', '210716_f3_clol', '210716_f6_clol', '210926_f2_gainmod', '211203_f0beyes_cl', '211207_f5beyes_natmov', '210923_f1b_gainmod', '211207_f1eyes_natmov', '210602_f3_natmov_eyes', '210923_f0_gainmod', '210314_f0_natmov', '210716_f2_clol', '210511_f3b_natmov', '210729_f6_clol', '210728_f2_clol', '210314_f1_natmov', '211118_f0_spont'}

[n_ring_neurons]
value = 74.0
interval = [48.5, 115.0]
n_fish = 3
moment = median
units = n
fids = all

[fit_anatom_dist_data]
value = 25.8
interval = [4.9, 83.7]
n_fish = 3
moment = median
units =
fids = all

[fit_anatom_dist_shuf]
value = 72.4
interval = [16.2, 210.0]
n_fish = 3
moment = median
units =
fids = all

[circ_corr_anat]
```

```
value = 0.549
interval = [0.298, 0.696]
n_fish = 3
moment = median
units =
fids = all

[mean_angle_turned]
value = 0.828
interval = [0.492, 1.28]
n_fish = 3
moment = median
units =
fids = all

[head_phase_slope_data]
value = -1.01
interval = [-1.36, -0.457]
n_fish = 3
moment = median
units =
fids = all

[head_phase_slope_shuffle]
value = -0.0169
interval = [-0.0624, 0.0343]
n_fish = 3
moment = median
units =
fids = all

[head_phase_slope_data vs head_phase_slope_shuffle]
s = 0.0
p_val = 1.17e-06
test = wilcoxon

[pcapop_variance_explained_r1pi]
value = 0.8
interval = [0.77, 0.836]
n_fish = 3
moment = median
units = fract.
fids = all

[pcapop_variance_explained_nonr1pi]
value = 0.46
interval = [0.411, 0.511]
n_fish = 3
moment = median
units = fract.
fids = all

[pcapop_variance_explained_r1pi vs pcapop_variance_explained_nonr1pi]
s = 0.0
p_val = 1.17e-06
test = wilcoxon

[heading_phase_corr]
value = -0.723
```

```
interval = [-0.863, -0.564]
n_fish = 3
moment = median
units =
fids = all

[pcatime_variance_explained_r1pi]
value = 0.858
interval = [0.827, 0.868]
n_fish = 3
moment = median
units = fract.
fids = all

[pcatime_variance_explained_nonr1pi]
value = 0.297
interval = [0.263, 0.324]
n_fish = 3
moment = median
units = fract.
fids = all

[pcatime_variance_explained_r1pi vs pcatime_variance_explained_nonr1pi]
s = 0.0
p_val = 1.17e-06
test = wilcoxon

[FWHM_activation_resampled_angle]
value = 2.91
interval = 0.115
n_fish = 3
moment = mean
units = rad
fids = all

[heading_phase_slope_gain0.5]
value = -1.77
interval = [-2.08, -1.21]
n_fish = 5
moment = median
units =
fids = ['210923_f0_gainmod', '210923_f1b_gainmod', '210924_f1_gainmod',
'210924_f2_gainmod', '210926_f2_gainmod']

[heading_phase_slope_gain1]
value = -0.931
interval = [-1.86, -0.696]
n_fish = 5
moment = median
units =
fids = ['210923_f0_gainmod', '210923_f1b_gainmod', '210924_f1_gainmod',
'210924_f2_gainmod', '210926_f2_gainmod']

[heading_phase_slope_gain2]
value = -1.27
interval = [-1.48, -0.584]
n_fish = 5
moment = median
units =
```

fids = ['210923_f0_gainmod', '210923_f1b_gainmod', '210924_f1_gainmod', '210924_f2_gainmod', '210926_f2_gainmod']

[heading_phase_slope_gain-1]
value = -0.646
interval = [-1.17, -0.426]
n_fish = 5
moment = median
units =
fids = ['210923_f0_gainmod', '210923_f1b_gainmod', '210924_f1_gainmod', '210924_f2_gainmod', '210926_f2_gainmod']

[heading_phase_slope_gain0.5 vs heading_phase_slope_gain1]
s = 3.0
p_val = 0.312
test = wilcoxon

[heading_phase_slope_gain0.5 vs heading_phase_slope_gain2]
s = 6.0
p_val = 0.812
test = wilcoxon

[heading_phase_slope_gain0.5 vs heading_phase_slope_gain-1]
s = 2.0
p_val = 0.188
test = wilcoxon

[heading_phase_slope_gain1 vs heading_phase_slope_gain2]
s = 5.0
p_val = 0.625
test = wilcoxon

[heading_phase_slope_gain1 vs heading_phase_slope_gain-1]
s = 5.0
p_val = 0.625
test = wilcoxon

[heading_phase_slope_gain2 vs heading_phase_slope_gain-1]
s = 4.0
p_val = 0.438
test = wilcoxon

[heading_phase_slope_closed_loop]
value = -0.579
interval = [-1.34, -0.257]
n_fish = 8
moment = median
units =
fids = ['210715_f5_clol', '210716_f2_clol', '210716_f3_clol', '210716_f6_clol', '210716_f7_clol', '210728_f1eyes_clol', '210728_f2_clol', '210729_f6_clol']

[heading_phase_slope_open_loop]
value = -0.595
interval = [-1.1, -0.37]
n_fish = 8
moment = median
units =
fids = ['210715_f5_clol', '210716_f2_clol', '210716_f3_clol', '210716_f6_clol', '210716_f7_clol', '210728_f1eyes_clol', '210728_f2_clol', '210729_f6_clol']

[heading_phase_slope_open_loop vs heading_phase_slope_closed_loop]
s = 18.0
p_val = 1.0
test = wilcoxon

[slope_cl]
value = -0.579
interval = [-1.34, -0.257]
n_fish = 8
moment = median
units =
fids = ['210715_f5_clol', '210716_f2_clol', '210716_f3_clol', '210716_f6_clol', '210716_f7_clol', '210728_f1eyes_clol', '210728_f2_clol', '210729_f6_clol']

[slope_ol]
value = -0.595
interval = [-1.1, -0.37]
n_fish = 8
moment = median
units =
fids = ['210715_f5_clol', '210716_f2_clol', '210716_f3_clol', '210716_f6_clol', '210716_f7_clol', '210728_f1eyes_clol', '210728_f2_clol', '210729_f6_clol']

[FWHM_activation_not_resampled (percent)]
value = 47.3
interval = 1.82
n_fish = 3
moment = mean
units = percent
fids = all

[eyes_fit_heading+gaze]
value = 0.209
interval = [0.173, 0.391]
n_fish = 7
moment = median
units =
fids = ['210601_f0_2dvr_eyes', '210602_f3_natmov_eyes', '210728_f1eyes_clol', '211203_f0beyes_cl', '211207_f1eyes_natmov', '211207_f3eyes_natmov', '211207_f5beyes_natmov']

[eyes_fit_gaze]
value = 0.0507
interval = [-0.0155, 0.125]
n_fish = 7
moment = median
units =
fids = ['210601_f0_2dvr_eyes', '210602_f3_natmov_eyes', '210728_f1eyes_clol', '211203_f0beyes_cl', '211207_f1eyes_natmov', '211207_f3eyes_natmov', '211207_f5beyes_natmov']

[eyes_fit_heading]
value = 0.348
interval = [0.139, 0.404]
n_fish = 7
moment = median
units =

fids = ['210601_f0_2dvr_eyes', '210602_f3_natmov_eyes', '210728_f1eyes_clol', '211203_f0beyes_cl', '211207_f1eyes_natmov', '211207_f3eyes_natmov', '211207_f5beyes_natmov']

[eyes_fit_heading+gaze vs eyes_fit_gaze]
s = 1.0
p_val = 0.0312
test = wilcoxon

[eyes_fit_heading+gaze vs eyes_fit_heading]
s = 9.0
p_val = 0.469
test = wilcoxon

[eyes_fit_gaze vs eyes_fit_heading]
s = 2.0
p_val = 0.0469
test = wilcoxon
