# Supplementary material for: Neural dynamics and architecture of the heading direction circuit in zebrafish
Source: Nat Neurosci. 2023 Apr 24;26(5):765–73. doi: 10.1038/s41593-023-01308-5 (PMC10166860; doi:10.1038/s41593-023-01308-5)
Supplement: Supplementary file 2 — Reporting Summary [file 41593_2023_1308_MOESM2_ESM.pdf]

nature portfolio

Corresponding author(s): Ruben Portugues

Last updated by author(s): Mar 9, 2023

Reporting Summary

Nature Portfolio wishes to improve the reproducibility of the work that we publish. This form provides structure for consistency and transparency in reporting. For further information on Nature Portfolio policies, see our Editorial Policies and the Editorial Policy Checklist.

Statistics

For all statistical analyses, confirm that the following items are present in the figure legend, table legend, main text, or Methods section.

n/a	Confirmed	
☐	☒	The exact sample size (*n*) for each experimental group/condition, given as a discrete number and unit of measurement
☐	☒	A statement on whether measurements were taken from distinct samples or whether the same sample was measured repeatedly
☐	☒	The statistical test(s) used AND whether they are one- or two-sided<br>*Only common tests should be described solely by name; describe more complex techniques in the Methods section.*
☐	☒	A description of all covariates tested
☐	☒	A description of any assumptions or corrections, such as tests of normality and adjustment for multiple comparisons
☐	☒	A full description of the statistical parameters including central tendency (e.g. means) or other basic estimates (e.g. regression coefficient) AND variation (e.g. standard deviation) or associated estimates of uncertainty (e.g. confidence intervals)
☐	☒	For null hypothesis testing, the test statistic (e.g. F, t, r) with confidence intervals, effect sizes, degrees of freedom and P value noted<br>*Give P values as exact values whenever suitable.*
☒	☐	For Bayesian analysis, information on the choice of priors and Markov chain Monte Carlo settings
☒	☐	For hierarchical and complex designs, identification of the appropriate level for tests and full reporting of outcomes
☐	☒	Estimates of effect sizes (e.g. Cohen's *d*, Pearson's *r*), indicating how they were calculated

Our web collection on statistics for biologists contains articles on many of the points above.

Software and code

Policy information about availability of computer code

Data collection	Light sheet imaging data was acquired using Sashimi 0.2.1 (https://github.com/portugueslab/sasihmi), Two photon data was acquired using Brunoise 0.1 (https://github.com/portugueslab/brunoise), Tail and eye tracking were acquired using Stytra 0.8.34 (https://github.com/portugueslab/stytra). The Stytra scripts for the control of the experimental stimuli will be shared together with the rest of the code.
Data analysis	All parts of the data analysis were performed using Python 3.7, and Python libraries for scientific computing, in particular Numpy 1.25.3, Scipy 1.9.1 and Scikit-learn 1.2.1. The Python environment required to replicate the analysis in the paper can be found in the paper code repository. All figures were produced using Matplotlib 3.7.1. All statistical tests used were non-parametric, either Mann-Whitney U test for unpaired comparisons (mannwhitneyu from scipy.stats) or Wilcoxon signed-rank test for paired comparisons (wilcoxon from scipy.stats). Imaging data was preprocessed using Suite2p 0.9.2. Behavioral data was analyzed with the bouter package 0.2.0 and DeepLabCut 2.0.

For manuscripts utilizing custom algorithms or software that are central to the research but not yet described in published literature, software must be made available to editors and reviewers. We strongly encourage code deposition in a community repository (e.g. GitHub). See the Nature Portfolio guidelines for submitting code & software for further information.

Data

Policy information about availability of data

All manuscripts must include a data availability statement. This statement should provide the following information, where applicable:

- Accession codes, unique identifiers, or web links for publicly available datasets
- A description of any restrictions on data availability
- For clinical datasets or third party data, please ensure that the statement adheres to our policy

All the source data used in the functional imaging analysis (raw dF/F traces, ROI maps/coordinates, behavioral traces, and stimulus log from Stytra) and for the anatomical observations (confocal/ two-photon stacks, EM skeletons) can be found here: https://doi.org/10.5281/zenodo.6847130

Human research participants

Policy information about studies involving human research participants and Sex and Gender in Research.

Reporting on sex and gender	N/A
Population characteristics	N/A
Recruitment	N/A
Ethics oversight	N/A

Note that full information on the approval of the study protocol must also be provided in the manuscript.

Field-specific reporting

Please select the one below that is the best fit for your research. If you are not sure, read the appropriate sections before making your selection.

☒ Life sciences ☐ Behavioural & social sciences ☐ Ecological, evolutionary & environmental sciences

For a reference copy of the document with all sections, see nature.com/documents/nr-reporting-summary-flat.pdf

Life sciences study design

All studies must disclose on these points even when the disclosure is negative.

Sample size	Sample sizes were not pre-calculated: they were based on previous experimental knowledge. This knowledge was collected in previous studies done in the lab (Portugues et. al. 2014, Dragomir et. al. 2020, Markov et. al. 2021).
Data exclusions	The method section reports a description of the experiment inclusion percentage, and a discussion on the rationale for the exclusion of some animals. With our strategy of detecting the r1π network we managed to do so in approximately 20-30% of the imaged animals. In the rest of the fish, sometimes behavior was just very sparse (a few swims over the entire experiment), or not very directional (only forward swims performed). In other fish, even if behavior was good the anticorrelation criterion could find only a handful of strongly anticorrelated neurons. Although those neurons were likely to be of the described network, as their activity state changed with the occurrence of directional swims, the low number of ROIs made it impossible to properly characterize their population dynamics. Finally, in some fish the rotatory dynamics was observable only in a small temporal interval of the experiment, and they were not included in the dataset.
Replication	The observations were consistently reproduced in experiments carried over several different clutches of fish, and have been replicated in two different setups (light-sheet microscope, two-photon microscope). Analyses were run independently by multiple investigators.
Randomization	Each animal was randomly assigned to an experimental protocol (out of the various protocols described in the MEthods section and in Fig S6). Randomization was not relevant for any other part of the study.
Blinding	No different groups of animals were used, and therefore no blinding procedure was employed.

Reporting for specific materials, systems and methods

We require information from authors about some types of materials, experimental systems and methods used in many studies. Here, indicate whether each material, system or method listed is relevant to your study. If you are not sure if a list item applies to your research, read the appropriate section before selecting a response.

Materials & experimental systems

n/a	Involved in the study
☒	☐ Antibodies
☒	☐ Eukaryotic cell lines
☒	☐ Palaeontology and archaeology
☐	☒ Animals and other organisms
☒	☐ Clinical data
☒	☐ Dual use research of concern

Methods

n/a	Involved in the study
☒	☐ ChIP-seq
☒	☐ Flow cytometry
☒	☐ MRI-based neuroimaging

Animals and other research organisms

Policy information about studies involving animals; ARRIVE guidelines recommended for reporting animal research, and Sex and Gender in Research

Laboratory animals	zebrafish (Danio rerio) from Tüpfel long fin (TL) strain, 6-9 dpf
Wild animals	This study did not involve wild animals
Reporting on sex	All experiments were conducted on 6-9 dpf larvae of yet undetermined sex
Field-collected samples	This study did not involve animals collected from the field
Ethics oversight	All procedures related to animal handling were conducted following protocols approved by the Technische Universität München and the Regierung von Oberbayern (TVA # 55-2-1-54-2532-101-12)

Note that full information on the approval of the study protocol must also be provided in the manuscript.
